# Supplementary material for: Development and validation of an LC-MS/MS method for determination of hydroxychloroquine, its two metabolites, and azithromycin in EDTA-treated human plasma
Source: PLoS One. 2021 Mar 5;16(3):e0247356. doi: 10.1371/journal.pone.0247356 (PMC7935301; doi:10.1371/journal.pone.0247356)

**Analyte Name:** AZM1

| Expected Concentration | Number of Values | APCI+ Calculated Concentration | % Accuracy | ESI+ Calculated Concentration | % Accuracy |
|---|---|---|---|---|---|
| 2 | 1 | 5.24 | 262.0 | 1.97 | 98.3 |
| 20 | 1 | 13.08 | 65.4 | 4.99 | 99.8 |
| 50 | 1 | 25.68 | 51.4 | 10.80 | 108.0 |
| 100 | 1 | 54.51 | 54.5 | 18.42 | 92.1 |
| 200 | 1 | 112.87 | 56.4 | 49.89 | 99.8 |
| 500 | 1 | 441.98 | 88.4 | 100.64 | 100.6 |
| 1000 | 1 | 1218.64 | 121.9 | 195.55 | 97.8 |
| Regression Equation | | y = 0.00549 x + -0.0252 (r = 0.9649) | | y = 0.00846 x + 0.00213 (r = 0.9992) | |

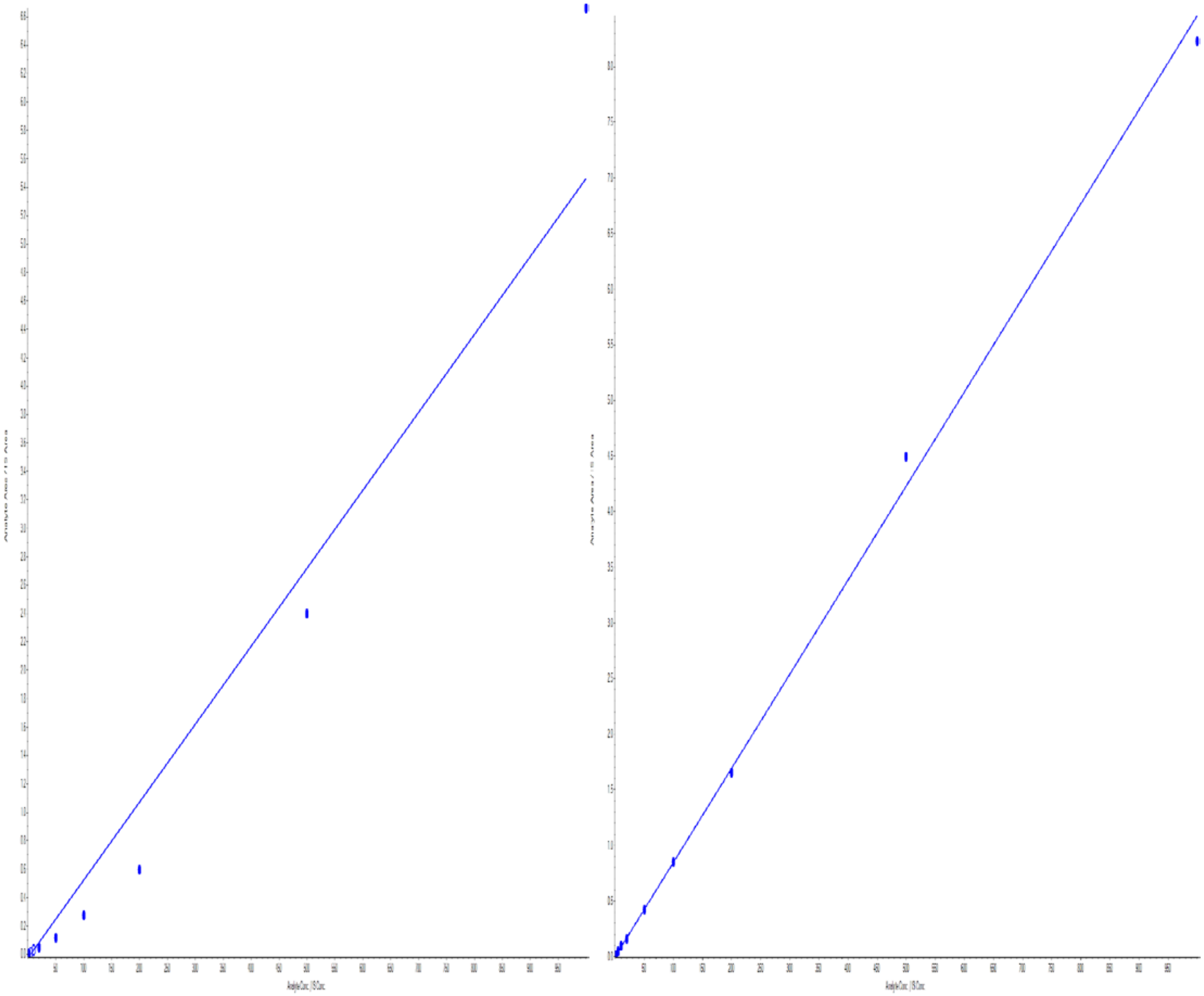

**Analyte Name:** HCQ

| Expected Concentration | Number of Values | APCI+ Calculated Concentration | % Accuracy | ESI+ Calculated Concentration | % Accuracy |
|---|---|---|---|---|---|
| 2 | 1 | 3.22 | 161.0 | 1.78 | 89.0 |
| 5 | 1 | 5.93 | 118.6 | 5.04 | 100.8 |
| 10 | 1 | 9.61 | 96.1 | 10.24 | 102.4 |
| 20 | 1 | 16.86 | 84.3 | 19.46 | 97.3 |
| 50 | 1 | 40.41 | 80.8 | 52.38 | 104.8 |
| 100 | 1 | 76.30 | 76.3 | 101.72 | 101.7 |
| 200 | 1 | 156.54 | 78.3 | 204.60 | 102.3 |
| 500 | 1 | 467.92 | 93.6 | 525.68 | 105.1 |
| 1000 | 1 | 1110.21 | 111.0 | 966.11 | 96.6 |
| Regression Equation | | y = 0.0445 x + -0.0741 (r = 0.9911) | | y = 0.0687 x + 0.0239 (r = 0.9992) | |

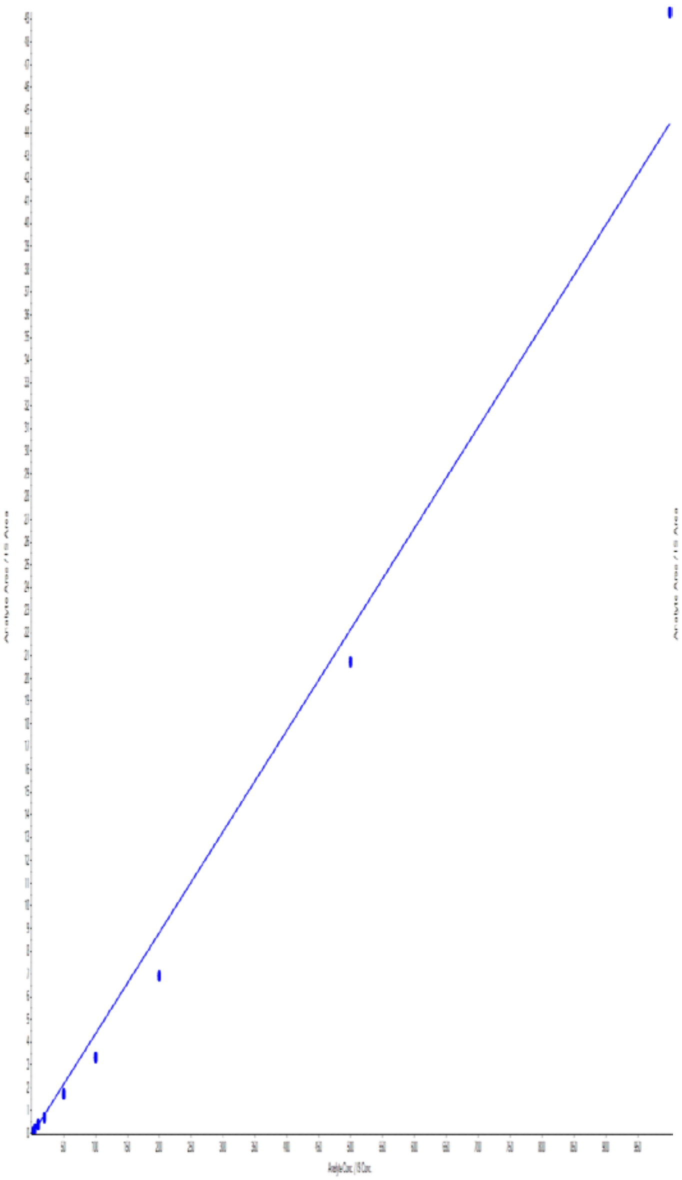

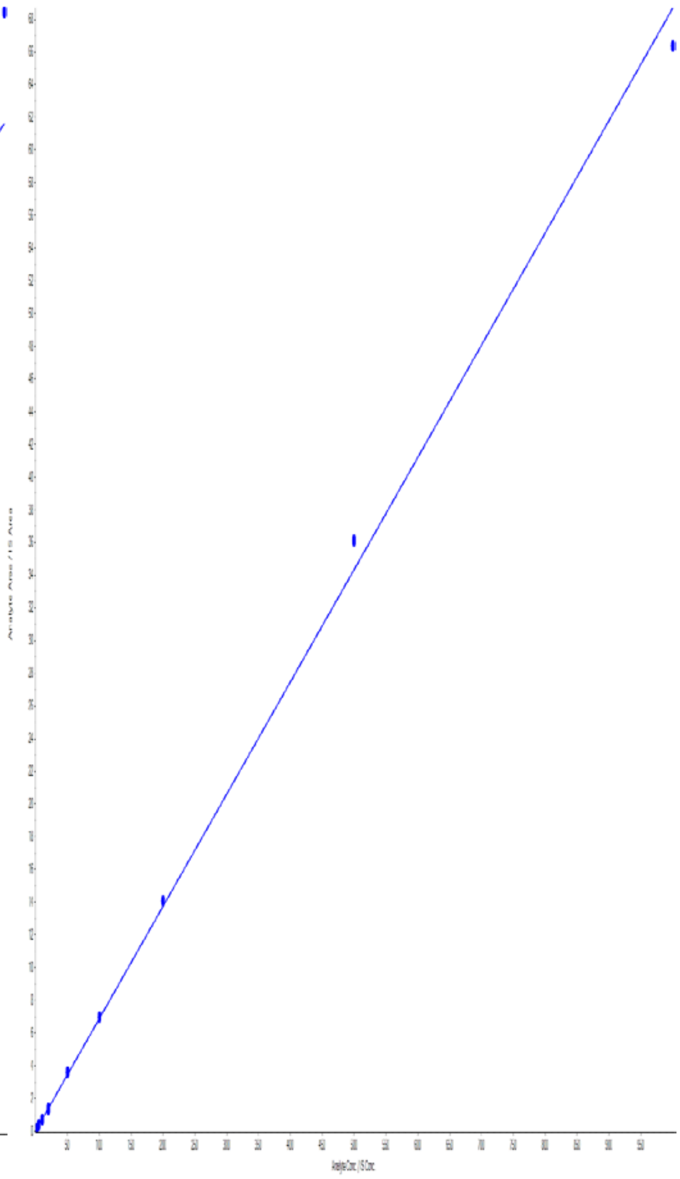

**Analyte Name:** DHCQ

| Expected Concentration | Number of Values | APCI+ Calculated Concentration | % Accuracy | ESI+ Calculated Concentration | % Accuracy |
|---|---|---|---|---|---|
| 1 | 1 | 1.06 | 106.2 | 0.96 | 95.9 |
| 2.5 | 1 | 2.32 | 92.7 | 2.60 | 104.2 |
| 5 | 1 | 4.97 | 99.4 | 4.85 | 97.1 |
| 10 | 1 | 9.66 | 96.6 | 9.72 | 97.2 |
| 25 | 1 | 25.78 | 103.1 | 25.69 | 102.8 |
| 50 | 1 | 51.19 | 102.4 | 52.22 | 104.4 |
| 100 | 1 | 103.01 | 103.0 | 98.95 | 99.0 |
| 250 | 1 | 237.17 | 94.9 | 249.06 | 99.6 |
| 500 | 1 | 508.34 | 101.7 | 499.44 | 99.9 |
| Regression Equation | | y = 0.107 x + 0.0188 (r = 0.9995) | | y = 0.104 x + 0.0136 (r = 0.9999) | |

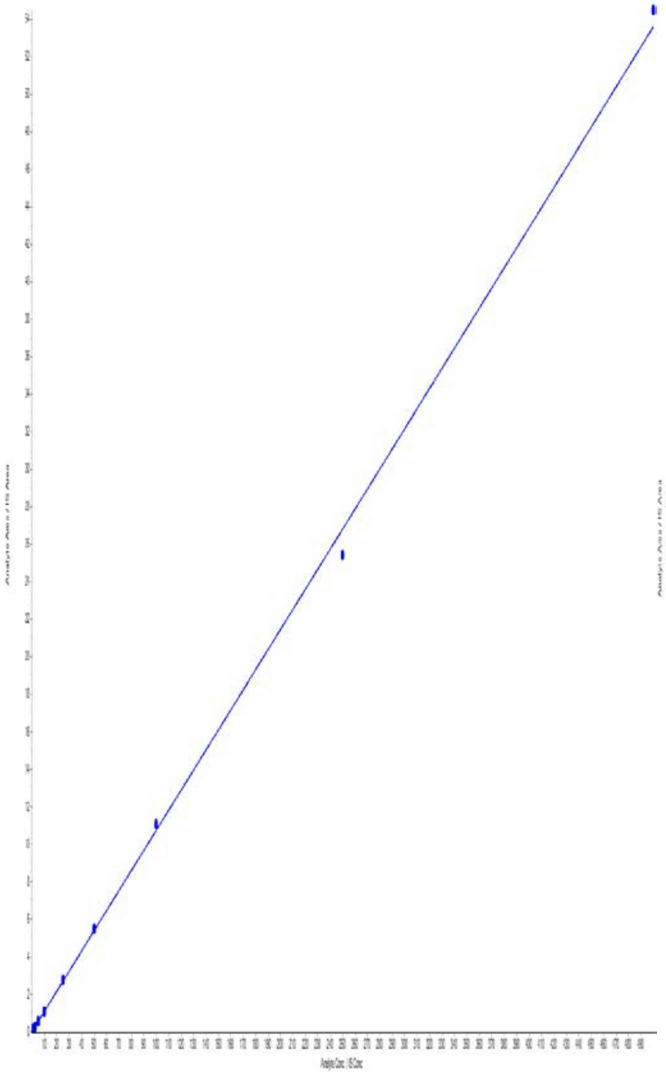

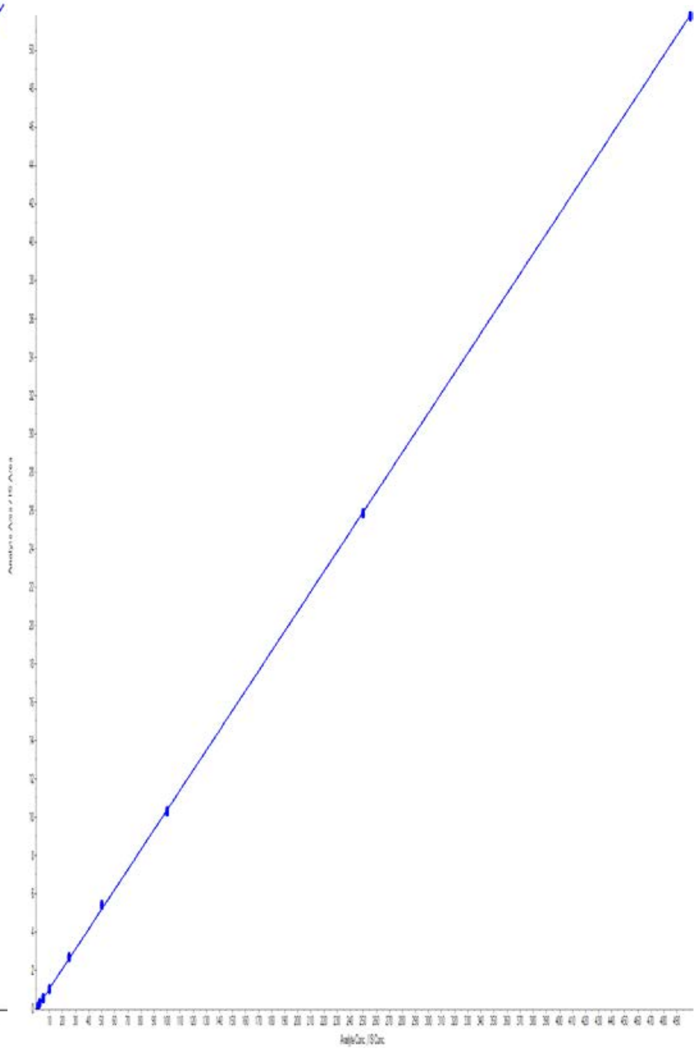

**Analyte Name:** BDCQ

| Expected Concentration | Number of Values | APCI+ Calculated Concentration | % Accuracy | ESI+ Calculated Concentration | % Accuracy |
|---|---|---|---|---|---|
| 0.5 | 1 | 0.64 | 128.8 | 0.51 | 101.9 |
| 1.25 | 1 | 1.07 | 85.3 | 1.23 | 98.7 |
| 2.5 | 1 | 2.43 | 97.2 | 2.70 | 108.0 |
| 5 | 1 | 5.31 | 106.3 | 4.93 | 98.7 |
| 12.5 | 1 | 11.18 | 89.4 | 12.56 | 100.5 |
| 25 | 1 | 24.68 | 98.7 | 22.65 | 90.6 |
| 50 | 1 | 44.45 | 88.9 | 49.63 | 99.3 |
| 125 | 1 | 131.52 | 105.2 | 128.42 | 102.7 |
| 250 | 1 | 250.46 | 100.2 | 249.11 | 99.6 |
| Regression Equation | | y = 0.12 x + -0.000467 (r = 0.9987) | | y = 0.135 x + 0.00314 (r = 0.9996) | |

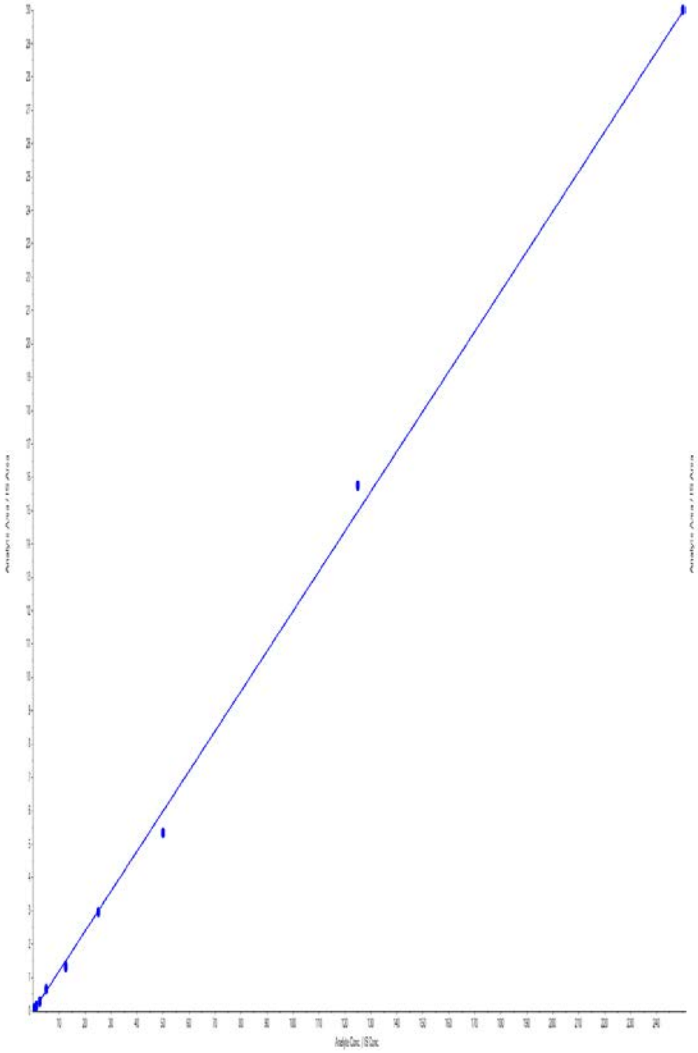

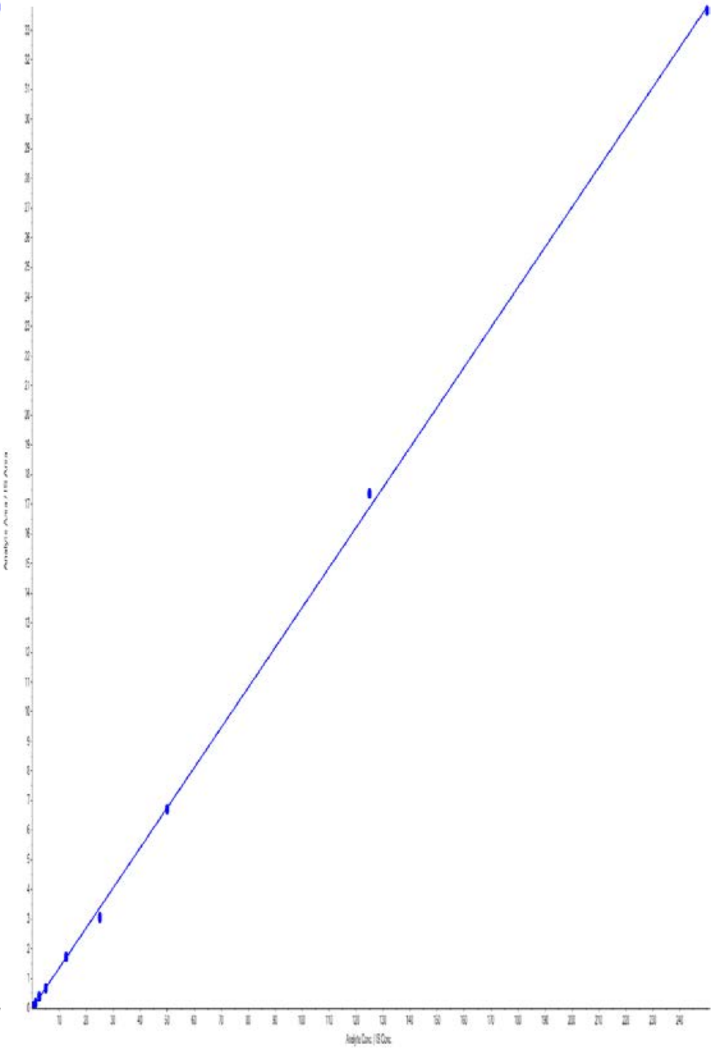

Supplement: S1 Fig — (PDF) [file pone.0247356.s001.pdf]
