# Supplementary material for: Development and validation of an LC-MS/MS method for determination of hydroxychloroquine, its two metabolites, and azithromycin in EDTA-treated human plasma
Source: PLoS One. 2021 Mar 5;16(3):e0247356. doi: 10.1371/journal.pone.0247356 (PMC7935301; doi:10.1371/journal.pone.0247356)

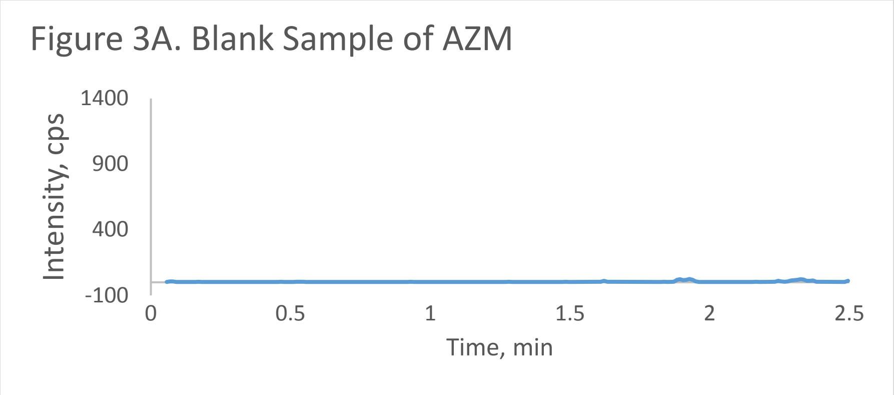
Figure 3A. Blank Sample of AZM
Intensity, cps
1400
900
400
-100
0
0.5
1
1.5
2
2.5
Time, min

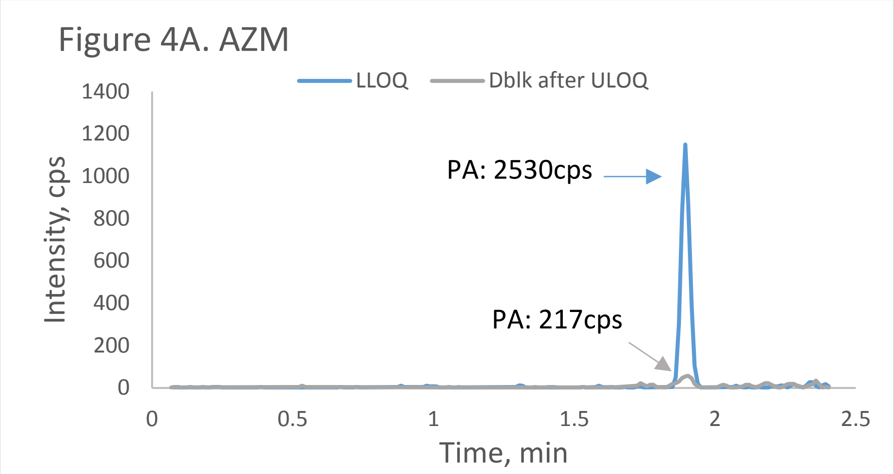
Figure 4A. AZM
LLOQ
Dblk after ULOQ
Intensity, cps
1400
1200
1000
800
600
400
200
0
PA: 2530cps
PA: 217cps
0
0.5
1
1.5
2
2.5
Time, min

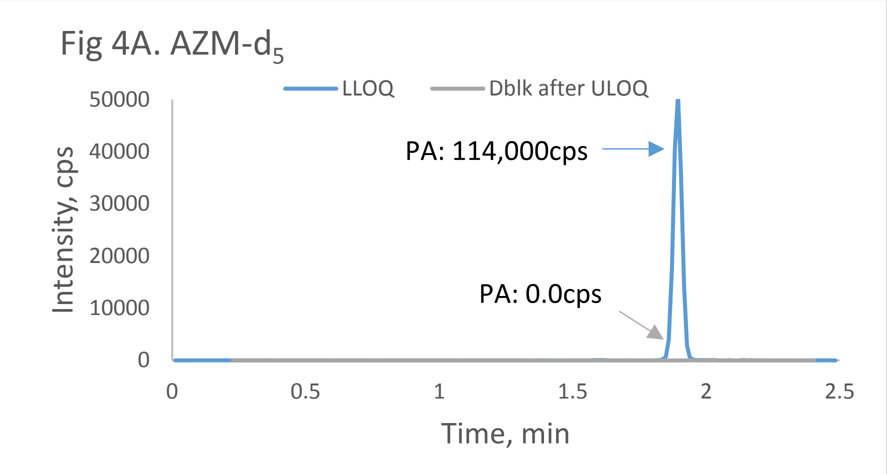
Fig 4A. AZM-d5
LLOQ
Dblk after ULOQ
Intensity, cps
50000
40000
30000
20000
10000
0
PA: 114,000cps
PA: 0.0cps
0
0.5
1
1.5
2
2.5
Time, min

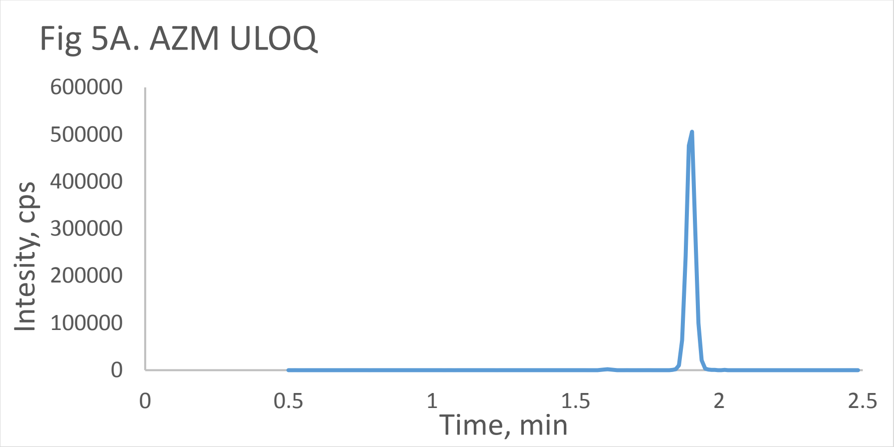
Fig 5A. AZM ULOQ
Intesity, cps
600000
500000
400000
300000
200000
100000
0
0
0.5
1
1.5
2
2.5
Time, min

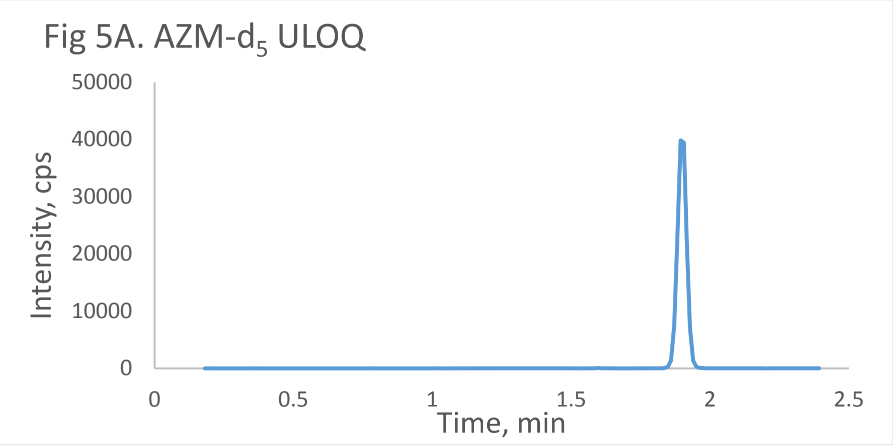
Fig 5A. AZM-d5 ULOQ
Intensity, cps
50000
40000
30000
20000
10000
0
0
0.5
1
1.5
2
2.5
Time, min

Fig 3B. Blank sample for HCQ

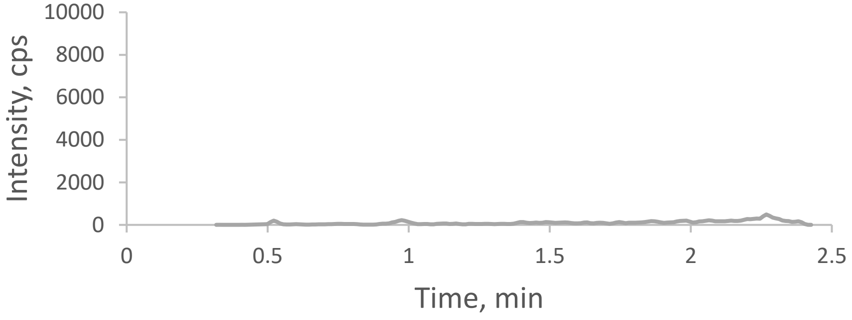

Fig. 4B HCQ

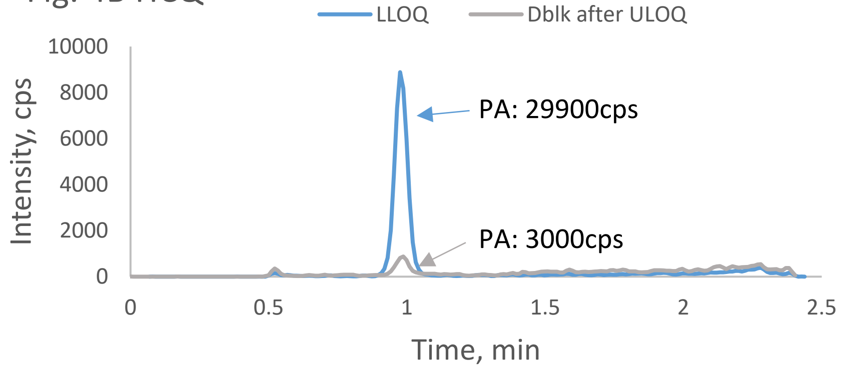

Fig. 4B HCQ-d_4

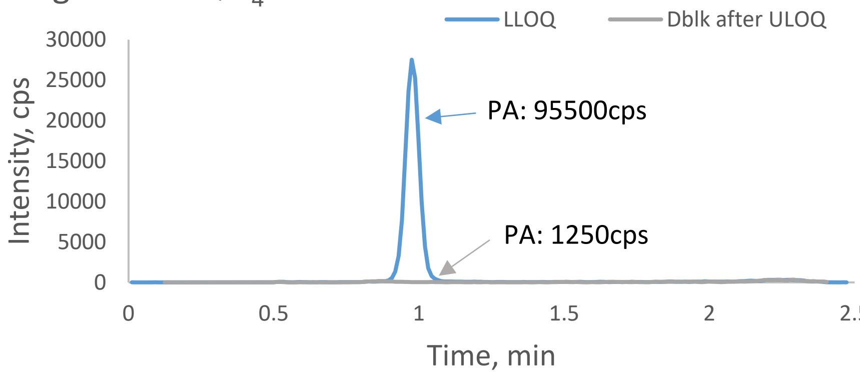

Fig. 5B HCQ ULOQ

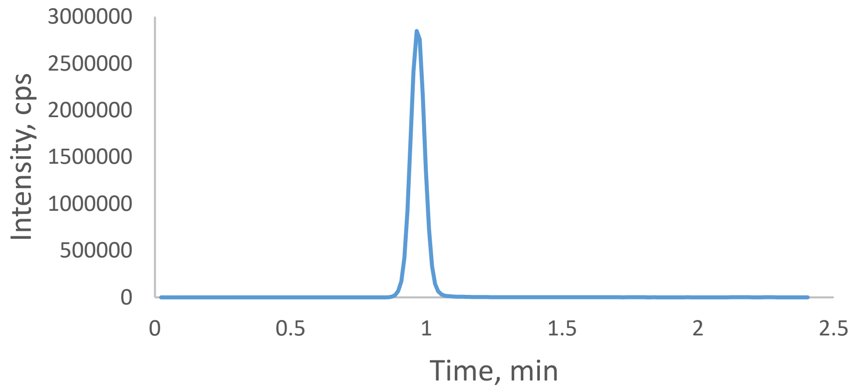

Fig 5B. HCQ-d_4 ULOQ

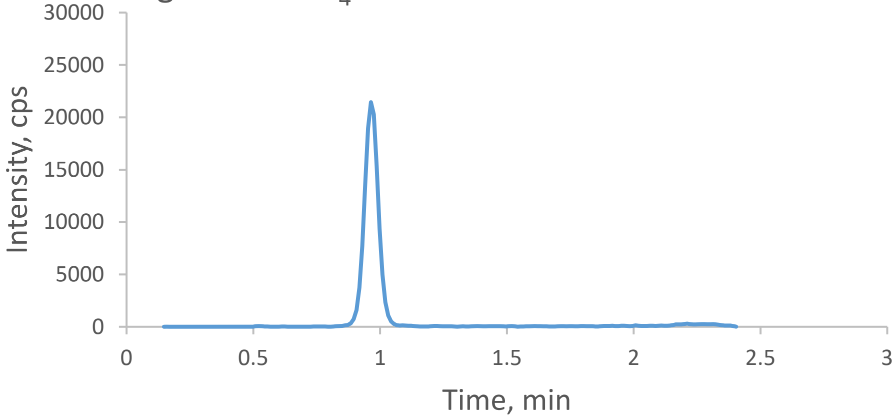

Figure 3C. Blank Sample of DHCQ

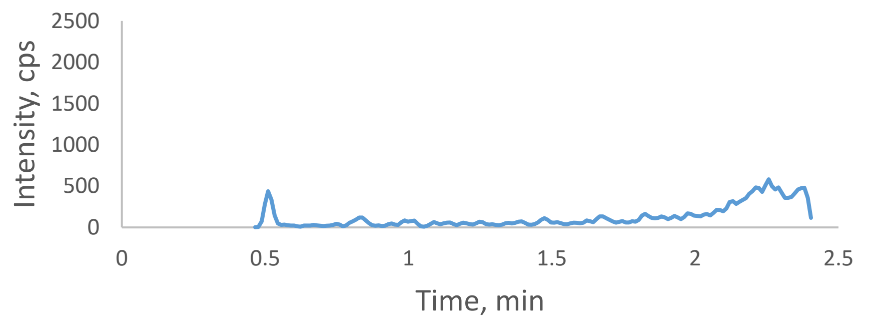

Fig 4C. DHCQ

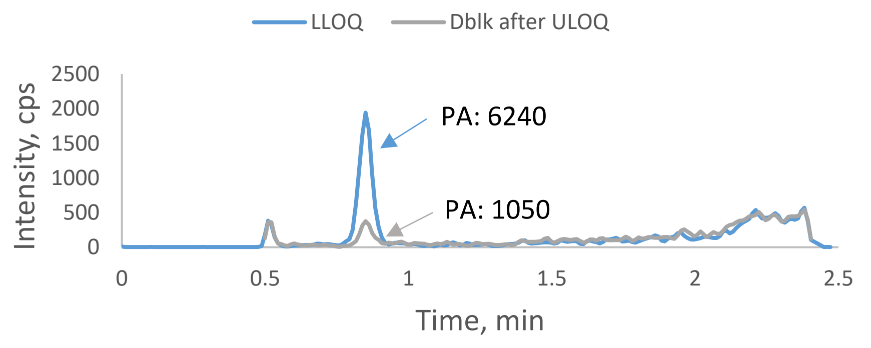

Figure 4C. DHCQ-d4

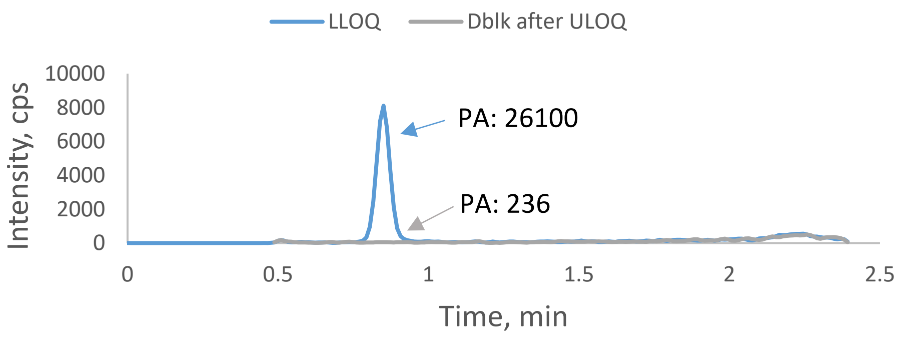

Figure 5C. DHCQ ULOQ

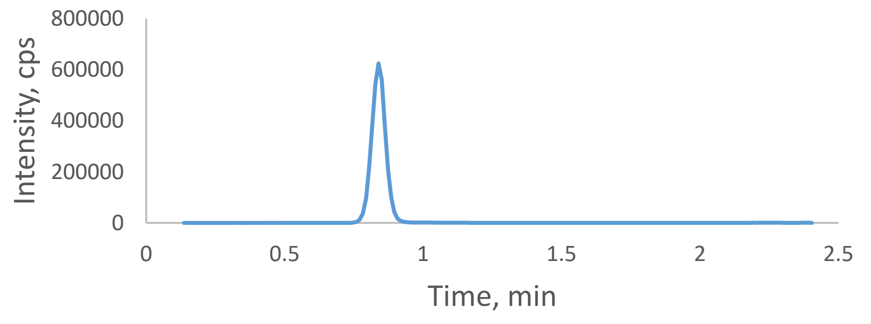

Fig 5C. DHCQ-d4 ULOQ

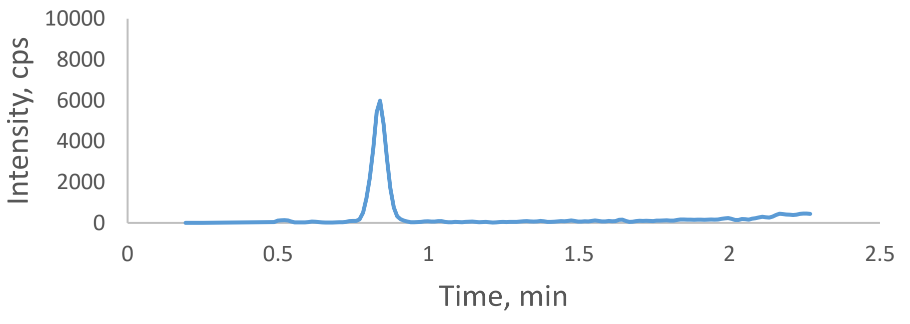

Figure 3D. Blank Sample for BDCQ

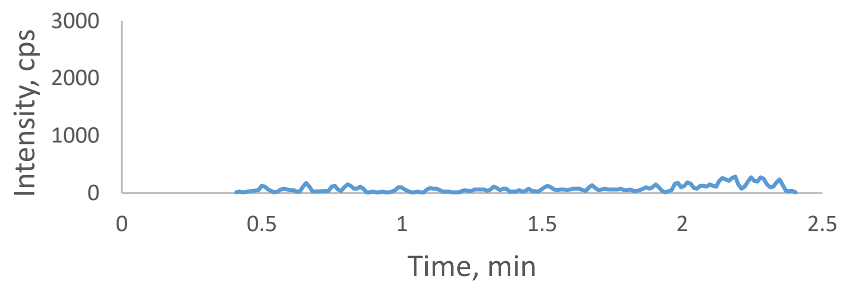

Fig 4D. BDCQ

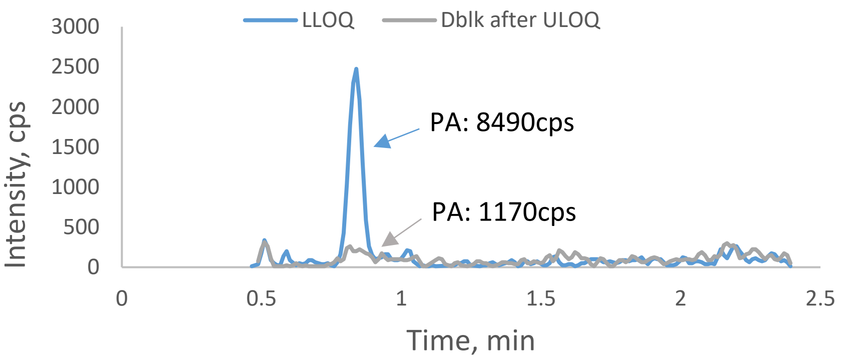

Fig 4D. BDCQ-d_4

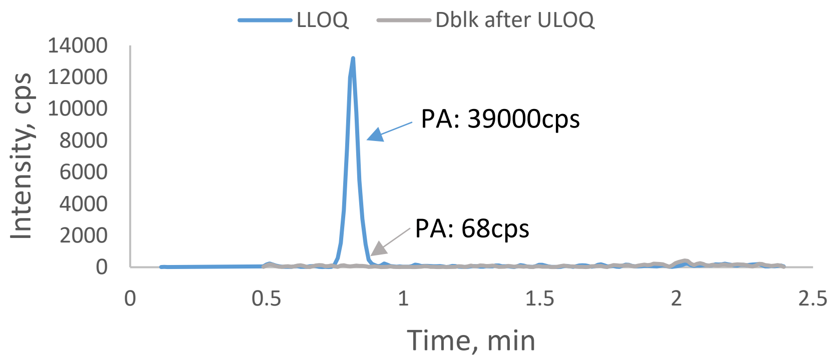

Fig 5D. BDCQ ULOQ Chromatogram

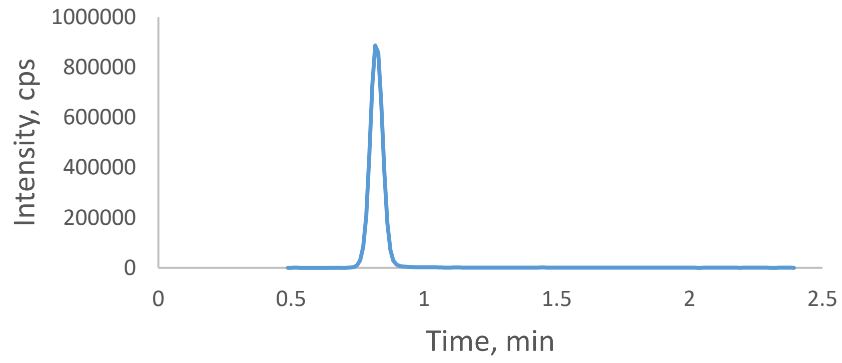

Fig 5D. BDCQ-d_4 from ULOQ

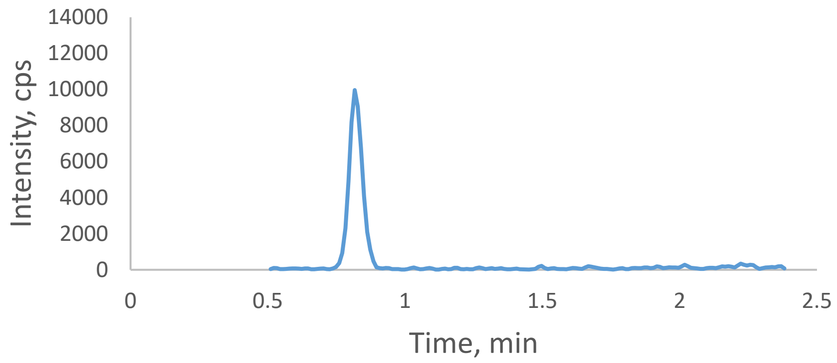

Supplement: S2 Fig — (PDF) [file pone.0247356.s002.pdf]
