# Supplementary material for: Development and validation of an LC-MS/MS method for determination of hydroxychloroquine, its two metabolites, and azithromycin in EDTA-treated human plasma
Source: PLoS One. 2021 Mar 5;16(3):e0247356. doi: 10.1371/journal.pone.0247356 (PMC7935301; doi:10.1371/journal.pone.0247356)
Supplement: S1 Table — (DOCX) [file pone.0247356.s003.docx]

**S1 Table. % Remaining of IS Peak Area Glass Vial vs Plastic Tube.**

| Glass Vial | AZM-d^5^ | HCQ-d^4^ | DHCQ-d^4^ | BDCQ-d^4^ |
| --- | --- | --- | --- | --- |
| RT, 6hr | 83 | 20 | 20 | 14 |
| RT, 23hr | 69 | 22 | 16 | 13 |
|  |  |  |  |  |
| Plastic tube | AZM-d^5^ | HCQ-d^4^ | DHCQ-d^4^ | BDCQ-d^4^ |
| RT, 6hr | 104 | 99 | 99 | 98 |
| RT, 23hr | 99 | 94 | 95 | 91 |

_Note: % Remaining vs frozen control at time zero, n=3_
