# Supplementary material for: Development and validation of an LC-MS/MS method for determination of hydroxychloroquine, its two metabolites, and azithromycin in EDTA-treated human plasma
Source: PLoS One. 2021 Mar 5;16(3):e0247356. doi: 10.1371/journal.pone.0247356 (PMC7935301; doi:10.1371/journal.pone.0247356)
Supplement: S2 Table — (PDF) [file pone.0247356.s004.pdf]

Table 5A: AZM Freeze/ Thaw Stability in Plasma

**Study Sample frozen Temperature:**			-70 C	**Matrix:**	Human plasma (K3EDTA)	
**Number of F/T cycles:**		4				
**Cycle**	**Date**	**Time Out**	**Time In**	**Duration (h:mm)**		
thaw cycle 1:	5/28/2020	6:47 AM	8:25 AM	1:38		
thaw cycle 2:	5/29/2020	11:00 AM	12:45 PM	1:45		
thaw cycle 3:	5/30/2020	7:00 AM	8:49 AM	1:49		
thaw cycle 4:	5/31/2020	7:25 AM	10:15 AM	2:50		

**Treated (Test QC)**	**Replicates**	**Low Test QC**	**High Test QC**	**Statistics**	**Low Test QC**	**High Test QC**
				**Nominal Conc.**	**6.00**	**800**
**RUN ID**				**Mean**	5.14	765
18	1	4.95	751	**SD**	0.36	23
	2	4.91	792	**%CV**	7.1	3.1
	3	5.56	752	**%Dev from Nominal Conc.**	-14	-4.4
				**n**	3	3

**Untreated (Control QC)**	**Replicates**	**Low Test QC**	**High Test QC**			
				**Nominal Conc.**	**6.00**	**800**
**RUN ID**				**Mean**	5.34	850
18	1	5.22	843	**SD**	0.23	18
	2	5.19	870	**%CV**	4.4	2.1
	3	5.61	836	**%Dev from Nominal Conc.**	-11	6.2
				**n**	3	3
				**% diff from control**	-3.7	-10

Table 5B: HCQ Freeze/ Thaw Stability in Plasma

**Study Sample frozen Temperature:**			-70 C	**Matrix:**	Human plasma (K3EDTA)	
**Number of F/T cycles:**		4				
**Cycle**	**Date**	**Time Out**	**Time In**	**Duration (h:mm)**		
thaw cycle 1:	5/28/2020	6:47 AM	8:25 AM	1:38		
thaw cycle 2:	5/29/2020	11:00 AM	12:45 PM	1:45		
thaw cycle 3:	5/30/2020	7:00 AM	8:49 AM	1:49		
thaw cycle 4:	5/31/2020	7:25 AM	10:15 AM	2:50		

**Treated (Test QC)**	**Replicates**	**Low Test QC**	**High Test QC**	**Statistics**	**Low Test QC**	**High Test QC**
				**Nominal Conc.**	**6.00**	**800**
**RUN ID**				**Mean**	5.78	784
18	1	5.79	769	**SD**	0.02	17
	2	5.76	802	**%CV**	0.4	2.1
	3	5.80	782	**%Dev from Nominal Conc.**	-3.6	-2.0
				**n**	3	3

**Untreated (Control QC)**	**Replicates**	**Low Test QC**	**High Test QC**			
				**Nominal Conc.**	**6.00**	**800**
**RUN ID**				**Mean**	6.34	793
18	1	6.46	786	**SD**	0.24	25
	2	6.50	821	**%CV**	3.8	3.1
	3	6.06	773	**%Dev from Nominal Conc.**	6	-0.8
				**n**	3	3
				**% diff from control**	-8.8	-1.1

Table 5C: DHCQ Freeze/ Thaw Stability in Plasma

Study Sample frozen Temperature:			-70 C		Matrix: Human plasma (K3EDTA)
Number of F/T cycles:		4			
Cycle	Date	Time Out	Time In	Duration (h:mm)	
thaw cycle 1:	5/28/2020	6:47 AM	8:25 AM	1:38	
thaw cycle 2:	5/29/2020	11:00 AM	12:45 PM	1:45	
thaw cycle 3:	5/30/2020	7:00 AM	8:49 AM	1:49	
thaw cycle 4:	5/31/2020	7:25 AM	10:15 AM	2:50	

Treated (Test QC)	Replicates	Low Test QC	High Test QC	Statistics	Low Test QC	High Test QC
				Nominal Conc.	3.00	400
RUN ID				Mean	2.92	373
18	1	2.83	379	SD	0.12	11
	2	3.06	360	%CV	4.1	3.0
	3	2.88	380	%Dev from Nominal Conc.	-2.6	-6.8
				n	3	3
**Untreated (Control QC)**	**Replicates**	**Low Test QC**	**High Test QC**	Nominal Conc.	3.00	400
RUN ID				Mean	3.32	394
18	1	3.45	366	SD	0.11	27
	2	3.24	397	%CV	3.4	6.8
	3	3.27	419	%Dev from Nominal Conc.	11	-1.5
				n	3	3
				% diff from control	-12	-5.3

Table 5D: BDCQ Freeze/ Thaw Stability in Plasma

Study Sample frozen Temperature:			-70 C		Matrix: Human plasma (K3EDTA)
Number of F/T cycles:		4			
Cycle	Date	Time Out	Time In	Duration (h:mm)	
thaw cycle 1:	5/28/2020	6:47 AM	8:25 AM	1:38	
thaw cycle 2:	5/29/2020	11:00 AM	12:45 PM	1:45	
thaw cycle 3:	5/30/2020	7:00 AM	8:49 AM	1:49	
thaw cycle 4:	5/31/2020	7:25 AM	10:15 AM	2:50	

Treated (Test QC)	Replicates	Low Test QC	High Test QC	Statistics	Low Test QC	High Test QC
				Nominal Conc.	1.50	200
RUN ID				Mean	1.49	189
18	1	1.58	191	SD	0.12	2
	2	1.36	187	%CV	7.7	1.1
	3	1.53	188	%Dev from Nominal Conc.	-0.7	-5.7
				n	3	3
**Untreated (Control QC)**	**Replicates**	**Low Test QC**	**High Test QC**	Nominal Conc.	1.50	200
RUN ID				Mean	1.60	197
18	1	1.57	200	SD	0.04	9
	2	1.64	204	%CV	2.2	4.5
	3	1.60	187	%Dev from Nominal Conc.	7	-1.5
				n	3	3
				% diff from control	-7.1	-4.2

Table 6A: Room Temperature Stability in Matrix for AZM

Time Samples Maintained at Room Temperature (hr):	66	Matrix:	Human plasma (K_3EDTA)
Room Temperature (°C):	22±3		
Container:	eppendorf tube		
Light exposure:	yes		

Treated	Replicates	Low Test QC	High Test QC	Statistics	Low Test QC	High Test QC
RUN ID	1	6.11	828	Nominal Conc.	**6.00**	**800**
14	2	5.82	757	Mean	5.98	801
	3	5.92	793	SD	0.13	33
	4	6.05	825	%CV	2.2	4.1
				%Dev from Nominal Conc.	-0.4	0.1
				n	4	4
**Untreated (Control QC)**	**Replicates**	**Low Test QC**	**High Test QC**	Nominal Conc.	**6.00**	**800**
				Mean	5.73	755
RUN ID	1	5.45	764	SD	0.26	23
14	2	5.79	729	%CV	4.5	3.0
	3	5.95	772	%Dev from Nominal Conc.	-4.5	-5.6
				n	3	3
% Difference between Treated (test QC) and Untreated (Control QC)					4.3	6.1

Table 6B: Room Temperature Stability in Matrix for HCQ

Time Samples Maintained at Room Temperature (hr):	66	Matrix:	Human plasma (K_3EDTA)
Room Temperature (°C):	22±3		
Container:	eppendorf tube		
Light exposure:	yes		

Treated	Replicates	Low Test QC	High Test QC	Statistics	Low Test QC	High Test QC
RUN ID	1	5.40	806	Nominal Conc.	**6.00**	**800**
14	2	5.09	736	Mean	5.46	786
	3	5.47	786	SD	0.32	36
	4	5.87	817	%CV	5.9	4.6
				%Dev from Nominal Conc.	-9.0	-1.7
				n	3	3
**Untreated (Control QC)**	**Replicates**	**Low Test QC**	**High Test QC**	Nominal Conc.	**6.00**	**800**
				Mean	5.59	784
RUN ID	1	5.95	816	SD	0.34	37
14	2	5.28	743	%CV	6.1	4.8
	3	5.53	794	%Dev from Nominal Conc.	-6.9	-2.0
				n	4	4
% Difference between Treated (test QC) and Untreated (Control QC)					-2.3	0.2

Table 6C: DHCQ Room Temperature Stability in Plasma

**Time Samples Maintained at Room Temperature (hr):**			66	**Matrix:**	Human plasma (K_3EDTA)	
**Room Temperature (°C):**	22±3					
**Container:**	eppendorf tube					
**Light exposure:**	yes					
**Treated**	**Replicates**	**Low Test QC**	**High Test QC**	**Statistics**	**Low Test QC**	**High Test QC**
**RUN ID**	1	2.60	363	**Nominal Conc.**	**3.00**	**400**
14	2	2.89	367	**Mean**	2.74	363
	3	2.73	380	**SD**	0.12	15
	4	2.73	343	**%CV**	4.3	4.2
				**%Dev from Nominal Conc.**	-8.7	-9.2
				**n**	4	4
**Untreated (Control QC)**	**5**	**Low Test QC**	**High Test QC**	**Nominal Conc.**	**3.00**	**400**
				**Mean**	2.96	390
**RUN ID**	1	2.82	390	**SD**	0.19	6.5
14	2	2.89	383	**%CV**	6.4	1.7
	3	3.18	396	**%Dev from Nominal Conc.**	-1.2	-2.6
				**n**	3	3
**% Difference between Treated (test QC) and Untreated (Control QC)**					-7.6	-6.8

Table 6D: BDCQ Room Temperature Stability in Plasma

**Time Samples Maintained at Room Temperature (hr):**			66	**Matrix:**	Human plasma (K_3EDTA)	
**Room Temperature (°C):**	22±3					
**Container:**	eppendorf tube					
**Light exposure:**	yes					
**Treated**	**Replicates**	**Low Test QC**	**High Test QC**	**Statistics**	**Low Test QC**	**High Test QC**
**RUN ID**	1	1.27	184	**Nominal Conc.**	**1.50**	**200**
14	2	1.28	176	**Mean**	1.30	177
	3	1.24	168	**SD**	0.07	7
	4	1.39	178	**%CV**	5.1	3.7
				**%Dev from Nominal Conc.**	-14	-12
				**n**	3	3
**Untreated (Control QC)**	**Replicates**	**Low Test QC**	**High Test QC**	**Nominal Conc.**	**1.50**	**200**
				**Mean**	1.48	188
**RUN ID**	1	1.55	201	**SD**	0.08	14
14	2	1.48	173	**%CV**	5.1	7.5
	3	1.40	191	**%Dev from Nominal Conc.**	-1.6	-5.8
				**n**	3	3
**% Difference between Treated (test QC) and Untreated (Control QC)**					-12	-6.3

Table 7A: AZM Reinjection Reproducibility in Prepared Samples

Reinjected Test QC Quantified off the Originally Injected Curve

**Temperature (°C):**	20±3		**Date/Time**
**Run ID:**	2	**Initial Analysis:**	4/17/20 9:07 PM
		**Reinjection Analysis:**	4/20/20 8:13 PM
		**Duration (hrs):**	**71**

Replicates	Low TEST QC	High TEST QC	Statistics	Low TEST QC	High TEST QC
1	5.38	812	**Nominal Conc.**	**6.00**	**800**
2	5.57	873	**Mean**	5.67	845
3	6.05	849	**SD**	0.35	31
			**%CV**	6.1	3.6
			**%Dev from Nominal Conc.**	-5.6	5.6
			**n**	3	3

Table 7B: HCQ Reinjection Reproducibility in Prepared Samples

Reinjected Test QC Quantified off the Originally Injected Curve

**Temperature (°C):**	20±3		**Date/Time**
**Run ID:**	2	**Initial Analysis:**	4/17/20 9:07 PM
		**Reinjection Analysis:**	4/20/20 8:13 PM
		**Duration (hrs):**	**71**

Replicates	Low TEST QC	High TEST QC	Statistics	Low TEST QC	High TEST QC
1	5.69	755	**Nominal Conc.**	**6.00**	**800**
2	5.80	644	**Mean**	5.81	718
3	5.95	756	**SD**	0.13	64
			**%CV**	2.2	9.0
			**%Dev from Nominal Conc.**	-3.1	-10
			**n**	3	3

Table 7C: DHCQ Reinjection Reproducibility in Prepared Samples

Reinjected Test QC Quantified off the Originally Injected Curve					
Temperature (°C):	20±3			Date/Time	
Run ID:	2		Initial Analysis:	4/17/20 9:07 PM	
			Reinjection Analysis:	4/20/20 8:13 PM	
			Duration (hrs):	**71**	
Replicates	Low TEST QC	High TEST QC	Statistics		
				Low TEST QC	High TEST QC
1	3.25	408	Nominal Conc.	**3.00**	**400**
2	3.11	412	Mean	3.16	410
3	3.11	410	SD	0.08	2
			%CV	2.6	0.5
			%Dev from Nominal Conc.	5.2	2.5
			n	3	3

Table 7D: BDCQ Reinjection Reproducibility in Prepared Samples

Reinjected Test QC Quantified off the Originally Injected Curve					
Temperature (°C):	20±3			Date/Time	
Run ID:	2		Initial Analysis:	4/17/20 9:07 PM	
			Reinjection Analysis:	4/20/20 8:13 PM	
			Duration (hrs):	71	
Replicates	Low TEST QC	High TEST QC	Statistics		
				Low TEST QC	High TEST QC
1	1.34	184	Nominal Conc.	**1.50**	**200**
2	1.59	200	Mean	1.50	187
3	1.56	177	SD	0.14	12
			%CV	9.1	6.3
			%Dev from Nominal Conc.	-0.2	-6.5
			n	3	3

Table 8A: AZM Long Term Stability in Plasma

**Duration**	4/17/2020	5/25/2020	**38**	days
**Run ID**	**15**		Storage:	-70 C
	**Control (fresh)**		Treated	
**Replicate**	**Low Test QC**	**High Test QC**	**Low Test QC**	**High Test QC**
1	5.45	886	6.10	838
2	5.95	909	5.89	869
3	6.33	863	6.40	871
**Statistics**				
**Nominal Conc.**	**6.00**	**800**	**6.00**	**800**
**Mean**	5.91	886	6.13	859
**SD**	0.44	47	0.26	33
**%CV**	7.5	5.3	4.2	3.9
**%Dev from Nominal Conc.**	-1.5	10.8	2.2	7.4
**% difference from Control**			3.7	-3.0
**n**	**3**	**3**	3	3

Table 8B: HCQ Long Term Stability in Plasma

**Duration**	4/17/2020	5/25/2020	**38**	days
**Run ID**	**15**		Storage:	-70 C
	**Control (fresh)**		Treated	
**Replicate**	**Low Test QC**	**High Test QC**	**Low Test QC**	**High Test QC**
1	5.21	763	5.20	810
2	5.04	789	5.24	814
3	5.15	756	5.16	746
**Statistics**				
**Nominal Conc.**	**6.00**	**800**	**6.00**	**800**
**Mean**	5.13	769	5.20	790
**SD**	0.09	21	0.04	32
**%CV**	1.7	2.7	0.8	4.0
**%Dev from Nominal Conc.**	-14	-3.8	-13	-1.3
**% difference from Control**			1.3	2.7
**n**	**3**	**3**	3	3

Table 8C: DHCQ Long Term Stability in Plasma

**Duration**	4/17/2020	5/25/2020	**38**	days
**Run ID**	**15**		Storage:	-70 C
	**Control (fresh)**		Treated	
**Replicate**	**Low Test QC**	**High Test QC**	**Low Test QC**	**High Test QC**
1	2.62	365	2.55	380
2	2.60	381	2.57	379
3	2.66	364	2.61	341
**Statistics**				
**Nominal Conc.**	**3.00**	**400**	**3.00**	**400**
**Mean**	2.63	370	2.58	367
**SD**	0.03	17	0.03	25
**%CV**	1.2	4.6	1.2	6.7
**%Dev from Nominal Conc.**	-12	-7.5	-14	-8.3
**% difference from Control**			-1.9	-0.9
**n**	**3**	**3**	3	3

Table 8D: BDCQ Long Term Stability in Plasma

**Duration**	4/17/2020	5/25/2020	**38**	days
**Run ID**	**15**		Storage:	-70 C
	**Control (fresh)**		Treated	
**Replicate**	**Low Test QC**	**High Test QC**	**Low Test QC**	**High Test QC**
1	1.56	181	1.25	184
2	1.28	194	1.25	188
3	1.21	191	1.47	162
**Statistics**				
**Nominal Conc.**	**1.50**	**200**	**1.50**	**200**
**Mean**	1.35	189	1.32	178
**SD**	0.19	8	0.13	16
**%CV**	14	4.2	9.6	8.9
**%Dev from Nominal Conc.**	-10	-5.7	-12	-11
**% difference from Control**			-2.0	-5.7
**n**	3	3	3	3

Table 9A: AZM Solution Stability

Stock Solution 2		Storage Time	Storage Temperature (°C)	Replicate	Freshly Prepared Solution stk3	OR Unexpired Stability Stock Solution	Stock Solution Stk2
					Peak Area		
		45 days	-70	1	902000		904000
Concentration:	2mg/ml			2	870000		856000
Solvent:	50% MeOH			3	956000		966000
Container:	1.5 eppendorf tube			**Mean**	**909333**		**908667**
Conc. Tested:	80 ng/mL			**SD**	**43466**		**55148**
Fresh Lot ID:	5.25.2020Stock			**%CV**	**4.8**		**6.1**
Stored Lot ID:	4.10.2020 Stk2			**%Difference**			**-0.1**
RUN ID:	15			**n**	**3**		**3**

Stock Solution 1		Storage Time	Storage Temperature (°C)	Replicate	Freshly Prepared Solution	OR Unexpired Stability Stock Solution	Stored Stability Stock Solution
					Peak Area		
		14hr	21-25	1		350000	326000
Concentration:	2mg/ml			2		343000	348000
Solvent:	MeOH			3		331000	362000
Container:	1.5 eppendorf tube			**Mean**		**341333**	**345333**
Conc. Tested:	50 ng/mL			**SD**		**9609**	**18148**
Fresh Lot ID:	4.10.2020Stock1 frozen -70C			**%CV**		**2.8**	**5.3**
Stored Lot ID:	4.24.2020 on bench overnight			**%Difference**			**1.2**
RUN ID:	11			**n**		**3**	**3**

Stock Solution		Storage Time	Storage Temperature (°C)	Replicate	Freshly Prepared Solution	OR Unexpired Stability Stock Solution	Stored Stability Stock Solution
					Peak Area		
		23hr	21-25	1		885000	905000
Concentration:	2mg/ml			2		911000	846000
Solvent:	50% MeOH			3		854000	864000
Container:	1.5 eppendorf tube			**Mean**		**883333**	**871667**
Conc. Tested:	80 ng/mL			**SD**		**28537**	**30238**
Fresh Lot ID:	5.25.2020Stock frozen -70C			**%CV**		**3.2**	**3.5**
Stored Lot ID:	05.30.2020 on bench overnight			**%Difference**			**-1.3**
RUN ID:	18			**n**		**3**	**3**

Stock Solution 3		Storage Time	Storage Temperature (°C)	Replicate	Freshly Prepared Solution	OR Unexpired Stability Stock Solution	Stored Stability Stock Solution
					Peak Area		
		6 days	21-25	1		353000.00	386000
Concentration:	0.5mg/ml			2		392000.00	363000
Solvent:	MeOH			3		378000.00	418000
Container:	1.5 eppendorf tube			**Mean**		**374333**	**389000**
Conc. Tested:	50 ng/mL			**SD**		**19757**	**27622**
Fresh Lot ID:	4.11.2020Stock_3USP frozen -70C			**%CV**		**5.3**	**7.1**
Stored Lot ID:	04.18.2020 on bench until 04.24.2020			**%Difference**			**3.9**
RUN ID:	10			**n**		**3**	**3**

Table 9A: AZM Solution Stability Continues

Working solution		Storage Time	Storage Temperature (°C)	Replicate	Freshly Prepared Solution	OR Unexpired Stability Stock Solution	Stored Stability Stock Solution
					Peak Area		
		36 days	-70	1	902000		854000
Concentration:	40 ug/ml			2	870000		910000
Solvent:	50%MeOH			3	956000		879000
Container:	1.5 eppendorf tube			**Mean**	**909333**		**881000**
Conc. Tested:	80 ng/mL			**SD**	**43466**		**28054**
Fresh Lot ID:	5.25.2020Stock			**%CV**	**4.8**		**3.2**
Stored Lot ID:	04.19.2020			**%Difference**			**-3.1**
RUN ID:	15			**n**	**3**		**3**

Working Solution		Storage Time	Storage Temperature (°C)	Replicate	Freshly Prepared Solution	OR Unexpired Stability Stock Solution	Stored Stability Stock Solution
					Peak Area		
		20 h	19-22	1		1540000	1410000
Concentration:	20 ug/mL			2		1490000	1510000
Solvent:	50%MeOH			3		1470000	1420000
Container:	1.5 eppendorf tube			**Mean**		**1500000**	**1446667**
Conc. Tested:	100 ng/mL			**SD**		**36056**	**55076**
Fresh Lot ID:	4.17.2020WK_STD			**%CV**		**2.4**	**3.8**
Stored Lot ID:	4.17.2020 on bench overnight			**%Difference**			**-3.6**
RUN ID:	10			**n**		**3**	**3**

Working Solution		Storage Time	Storage Temperature (°C)	Replicate	Freshly Prepared Solution	OR Unexpired Stability Stock Solution	Stored Stability Stock Solution
					Peak Area		
		8 days	19-22	1		261000	273000
Concentration:	40 ug/mL			2		251000	231000
Solvent:	50%MeOH			3		240000	224000
Container:	1.5 eppendorf tube			**Mean**		**250667**	**242667**
Conc. Tested:	50 ng/mL			**SD**		**10504**	**26502**
Fresh Lot ID:	7.12.2020WK_QC			**%CV**		**4.2**	**10.9**
Stored Lot ID:	7.12.2020 WK on bench until 7.20.2020			**%Difference**			**-3.2**
RUN ID:	19			**n**		**3**	**3**

IS Working Solution		Storage Time	Storage Temperature (°C)	Replicate	frozen Solution (Control)	glass (1.5dr) 16x50mm	plastic (1.5mL Eppendorf tube)
					Peak Area		
		6 hr	21-25	1	7.15E+04	6.12E+04	76600
Concentration:	100 ng/mL			2	7.62E+04	6.23E+04	73400
Solvent:	50% MeOH			3	7.41E+04	6.17E+04	80800
Container:	Eppendorf tube			**Mean**	**73933**	**61733**	**76933**
Conc. Tested:	10 ng/mL			**SD**	**2354**	**551**	**3711**
Fresh Lot ID:	Apr 19, 2020 freeze			**%CV**	**3.2**	**0.9**	**4.8**
Stored Lot ID:	Apr 23, 2020 Wk on bench			**%Difference**		**-17**	**4.1**
RUN ID:	10			**n**		**3**	**3**

IS Working Solution		Storage Time	Storage Temperature (°C)	Replicate	frozen Solution (Control)	glass (1.5dr) 16x50mm	plastic (1.5mL Eppendorf tube)
					Peak Area		
		23 hr	21-25	1	76100	49000	70800
Concentration:	100 ng/mL			2	71100	50900	75300
Solvent:	50% MeOH			3	75400	54000	77700
Container:	Eppendorf tube			**Mean**	**74200**	**51300**	**74600**
Conc. Tested:	10 ng/mL			**SD**	**2707**	**2524**	**3503**
Fresh Lot ID:	Apr 19, 2020 freeze			**%CV**	**3.6**	**4.9**	**4.7**
Stored Lot ID:	Apr 23, 2020 Wk on bench			**%Difference**		**-31**	**0.54**
RUN ID:	10			**n**		**3**	**3**

Table 9B: HCQ Solution Stability

Stock Solution		Storage Time	Storage Temperature (^oC)	Replicate	Freshly Prepared Solution	OR Unexpired Stability Stock Solution	Stored Stability Stock Solution
					Peak Area		
		63 days	-70	1	9930000		10600000
**Concentration:**	2mg/ml			2	10100000		10500000
**Solvent:**	water			3	10000000		10900000
**Container:**	1.5 eppendorf tube			**Mean**	**10010000**		**10666667**
**Conc. Tested:**	80 ng/mL			**SD**	**85440**		**208167**
**Fresh Lot ID:**	5.25.2020Stock_AK			**%CV**	**0.85**		**2.0**
**Stored Lot ID:**	03.23.2020			**%Difference**			**6.6**
**RUN ID:**	15			**n**	**3**		**3**

Stock Solution		Storage Time	Storage Temperature (^oC)	Replicate	Freshly Prepared Solution	OR Unexpired Stability Stock Solution	Stored Stability Stock Solution
					Peak Area		
		5 days	21-25	1		7860000.00	8200000
**Concentration:**	2mg/ml			2		7930000.00	8250000
**Solvent:**	water			3		8140000.00	7850000
**Container:**	1.5 eppendorf tube			**Mean**		**7976667**	**8100000**
**Conc. Tested:**	80 ng/mL			**SD**		**145717**	**217945**
**Fresh Lot ID:**	5.25.2020Stock freeze			**%CV**		**1.83**	**2.69**
**Stored Lot ID:**	05.25.2020 stock on bench until 05.30			**%Difference**			**1.55**
**RUN ID:**	17			**n**			**3**

Working Solution		Storage Time	Storage Temperature (^oC)	Replicate	Freshly Prepared Solution	OR Unexpired Stability Stock Solution	Stored Stability Stock Solution
					Peak Area		
		64 days	-70	1		7860000.00	7780000
**Concentration:**	20 ug/mL			2		7930000.00	8010000
**Solvent:**	water			3		8140000.00	7800000
**Container:**	1.5 eppendorf tube			**Mean**		**7976667**	**7863333**
**Conc. Tested:**	80 ng/mL			**SD**		**145717**	**127410**
**Fresh Lot ID:**	5.25.2020Stock_1USP			**%CV**		**1.83**	**1.62**
**Stored Lot ID:**	03.27.2020 at -70C until 05.30.2020			**%Difference**			**-1.42**
**RUN ID:**	17			**n**		**3**	**3**

Working Solution		Storage Time	Storage Temperature (^oC)	Replicate	Freshly Prepared Solution	OR Unexpired Stability Stock Solution	Stored Stability Stock Solution
					Peak Area		
		12 h	19-22	1	66916		63456
**Concentration:**	10 ug/mL			2	66110		63681
**Solvent:**	water			3	66226		63626
**Container:**	1.5 eppendorf tube			**Mean**	**66417**		**63588**
**Conc. Tested:**	10 ug/mL			**SD**	**436**		**117**
**Fresh Lot ID:**	4.04.2020Stock_1USP			**%CV**	**0.66**		**0.18**
**Stored Lot ID:**	4.03.2020 on bench overnight			**%Difference**			**-4.26**
**RUN ID:**	UPLC-UV 4.04.2020			**n**		**0**	**3**

Table 9B: HCQ Solution Stability Continues

Working Solution		Storage Time	Storage Temperature (°C)	Replicate	Freshly Prepared Solution	OR Unexpired Stability Stock Solution	Stored Stability Stock Solution
					Peak Area		
		8 days	19-22	1		4960000	5000000
Concentration:	40 ug/mL			2		5000000	4760000
Solvent:	50%MeOH			3		4980000	4670000
Container:	1.5 eppendorf tube			**Mean**	**#DIV/0!**	**4980000**	**4810000**
Conc. Tested:	50 ng/mL			**SD**	**#DIV/0!**	**20000**	**170587**
Fresh Lot ID:	7.12.2020WK_QC			**%CV**	**#DIV/0!**	**0.40**	**3.55**
Stored Lot ID:	7.12.2020 WK on bench until 7.20.2020			**%Difference**			**-3.41**
RUN ID:	19			**n**		**3**	**3**

IS Working Solution		Storage Time	Storage Temperature (°C)	Replicate	frozen Solution (Control)	glass (1.5dr) 16x50mm	plastic (1.5mL Eppendorf tube)
					Peak Area		
		6 hr	21-25	1	65800	13100	64000
Concentration:	40 ng/mL			2	62700	12800	63000
Solvent:	50% MeOH			3	63500	13200	64300
Container:	Eppendorf tube			**Mean**	**64000**	**13033**	**63767**
Conc. Tested:	4 ng/mL			**SD**	**1609**	**208**	**681**
Fresh Lot ID:	Apr 19, 2020 freeze			**%CV**	**2.5**	**1.6**	**1.1**
Stored Lot ID:	Apr 23, 2020 Wk on bench			**%Difference**		**-80**	**-0.36**
RUN ID:	10			**n**		**3**	**3**

IS Working Solution		Storage Time	Storage Temperature (°C)	Replicate	frozen Solution (Control)	glass (1.5dr) 16x50mm	plastic (1.5mL Eppendorf tube)
					Peak Area		
		23 hr	21-25	1	76300	16900	73700
Concentration:	40 ng/mL			2	78300	16700	71600
Solvent:	50% MeOH			3	75400	16300	70900
Container:	Eppendorf tube			**Mean**	**76667**	**16633**	**72067**
Conc. Tested:	4 ng/mL			**SD**	**1484**	**306**	**1457**
Fresh Lot ID:	Apr 19, 2020 freeze			**%CV**	**1.9**	**1.8**	**2.0**
Stored Lot ID:	Apr 23, 2020 Wk on bench			**%Difference**		**-78**	**-6.0**
RUN ID:	10			**n**		**3**	**3**

Table 9C: DHCQ Solution Stability

Stock Solution		Storage Time	Storage Temperature (°C)	Replicate	Freshly Prepared Solution (stk3)	OR Unexpired Stability Stock Solution	Stored Stability Stock Solution (stk2)
					Peak Area		
		1day	-70	1	354987		326470
**Concentration:**	1mg/ml			2	361490		340801
**Solvent:**	Methanol			3	369285		339790
**Container:**	1.5 eppendorf tube			**Mean**	**361921**		**335687**
**Conc. Tested:**	20 ug/mL			**SD**	**7159**		**7998**
**Fresh Lot ID:**	4.17.2020Stock_Stk3			**%CV**	**2.0**		**2.4**
**Stored Lot ID:**	04.16.2020			**%Difference**			**-7.2**
**RUN ID:**	Compare two stocks (Stock 2 QC and 3 STD)			**n**	**3**		**3**

Stock Solution		Storage Time	Storage Temperature (°C)	Replicate	Freshly Prepared Solution	OR Unexpired Stability Stock Solution	Stored Stability Stock Solution
					Peak Area		
		14h	21-25	1		1820000.00	1810000
**Concentration:**	1 mg/ml			2		1780000.00	1880000
**Solvent:**	Methanol			3		1790000.00	1960000
**Container:**	1.5 eppendorf tube			**Mean**		**1796667**	**1883333**
**Conc. Tested:**	50 ng/mL			**SD**		**20817**	**75056**
**Fresh Lot ID:**	4.16.2020Stk freeze at -70 C			**%CV**		**1.2**	**4.0**
**Stored Lot ID:**	04.24.2020 on bench overnight			**%Difference**			**4.8**
**RUN ID:**	11			**n**		**3**	**3**

Working Solution 1		Storage Time	Storage Temperature (°C)	Replicate	Freshly Prepared Solution	OR Unexpired Stability Stock Solution	Stored Stability Stock Solution
					Peak Area		
		1 days	-70	1	354987		334465
**Concentration:**	50 ug/mL			2	361490		334057
**Solvent:**	50%MeOH			3	369285		333433
**Container:**	1.5 eppendorf tube			**Mean**	**361921**		**333985**
**Conc. Tested:**	20 ug/mL			**SD**	**7159**		**520**
**Fresh Lot ID:**	4.17.2020Wk_Stk3			**%CV**	**2.0**		**0.16**
**Stored Lot ID:**	04.16.2020			**%Difference**			**-7.7**
**RUN ID:**	UPLC-UV 4.17.2020			**n**	**3**		**3**

Working Solution 1		Storage Time	Storage Temperature (°C)	Replicate	Freshly Prepared Solution	OR Unexpired Stability Stock Solution	Stored Stability Stock Solution
					Peak Area		
		17 h	19-22	1		334465	335955
**Concentration:**	50 ug/mL			2		334057	335870
**Solvent:**	50% MeOH			3		333433	336419
**Container:**	1.5 eppendorf tube			**Mean**		**333985**	**336081**
**Conc. Tested:**	20 ug/mL			**SD**		**520**	**296**
**Fresh Lot ID:**	4.16.2020Wk_Frezze-70C			**%CV**		**0.16**	**0.09**
**Stored Lot ID:**	4.16.2020 on bench overnight			**%Difference**			**0.63**
**RUN ID:**	UPLC-UV 4.17.2020			**n**		**3**	**3**

Table 9C: DHCQ Solution Stability Continues

Working Solution 2		Storage Time	Storage Temperature (^oC)	Replicate	Freshly Prepared Solution	OR Unexpired Stability Stock Solution	Stored Stability Stock Solution
					Peak Area		
		8 days	19-22	1		817000	812000
**Concentration:**	20 ug/mL			2		799000	773000
**Solvent:**	50% MeOH			3		829000	790000
**Container:**	1.5 eppendorf tube			**Mean**		**815000**	**791667**
**Conc. Tested:**	25 ng/mL			**SD**		**15100**	**19553**
**Fresh Lot ID:**	7.12.2020WK_QC			**%CV**		**1.85**	**2.47**
**Stored Lot ID:**	7.12.2020 WK on bench until 7.20.2020			**%Difference**			**-2.86**
**RUN ID:**	19			**n**		**3**	**3**

Working Solution 2		Storage Time	Storage Temperature (^oC)	Replicate	Freshly Prepared Solution	OR Unexpired Stability Stock Solution	Stored Stability Stock Solution
					Peak Area		
		20 h	19-22	1		166110	168372
**Concentration:**	20 ug/mL			2		165560	167726
**Solvent:**	50% MeOH			3		164959	167471
**Container:**	1.5 eppendorf tube			**Mean**		**165543**	**167856**
**Conc. Tested:**	10 ug/mL			**SD**		**576**	**464**
**Fresh Lot ID:**	4.17.2020Wk_Frezze-70 C			**%CV**		**0.35**	**0.28**
**Stored Lot ID:**	4.17.2020 on bench overnight			**%Difference**			**1.4**
**RUN ID:**	UPLC-UV 4.18.2020			**n**		**3**	**3**

IS Working Solution		Storage Time	Storage Tempera ture (^oC)	Replicate	frozen Solution (Control)	glass (1.5dr) 16x50mm	plastic (1.5mL Eppendorf tube)
					Peak Area		
		6 hr	21-25	1	18800	3650	18500
**Concentration:**	20 ng/mL			2	19600	3760	17800
**Solvent:**	50% MeOH			3	17600	3950	19500
**Container:**	Eppendorf tube			**Mean**	**18667**	**3787**	**18600**
**Conc. Tested:**	2 ng/mL			**SD**	**1007**	**152**	**854**
**Fresh Lot ID:**	Apr 19, 2020 freeze			**%CV**	**5.4**	**4.0**	**4.6**
**Stored Lot ID:**	Apr 23, 2020 Wk on bench			**%Difference**		**-80**	**-0.36**
**RUN ID:**	10			**n**		**3**	**3**

IS Working Solution		Storage Time	Storage Temperature (^oC)	Replicate	frozen Solution (Control)	glass (1.5dr) 16x50mm	plastic (1.5mL Eppendorf tube)
					Peak Area		
		23 hr	21-25	1	23900	3540	23200
**Concentration:**	20 ng/mL			2	23500	4000	22500
**Solvent:**	50% MeOH			3	24200	3800	22100
**Container:**	Eppendorf tube			**Mean**	**23867**	**3780**	**22600**
**Conc. Tested:**	2 ng/mL			**SD**	**351**	**231**	**557**
**Fresh Lot ID:**	Apr 19, 2020 freeze			**%CV**	**1.5**	**6.1**	**2.5**
**Stored Lot ID:**	Apr 23, 2020 Wk on bench			**%Difference**		**-84**	**-5.3**
**RUN ID:**	10			**n**		**3**	**3**

Table 9D: BDCQ Solution Stability

Stock Solution 1		Storage Time	Storage Temperature (^oC)	Replicate	Freshly Prepared Solution	OR Unexpired Stability Stock Solution	Stored Stability Stock Solution
					Peak Area		
		6 days	-70	1	406951		376297
**Concentration:**	1mg/ml			2	405946		368872
**Solvent:**	Methanol			3	405586		367959
**Container:**	1.5 eppendorf tube			**Mean**	**406161**		**371043**
**Conc. Tested:**	20 ug/mL			**SD**	**707**		**4573**
**Fresh Lot ID:**	4.16.2020Stock2			**%CV**	**0.17**		**1.2**
**Stored Lot ID:**	04.10.2020			**%Difference**			**-8.6**
**RUN ID:**	UPLC-UV 4.16.2020			**n**	**3**		**3**

Stock Solution 1		Storage Time	Storage Temperature (^oC)	Replicate	Freshly Prepared Solution	OR Unexpired Stability Stock Solution	Stored Stability Stock Solution
					Peak Area		
		20h	19-22	1		368596	369742
**Concentration:**	1mg/ml			2		368898	369823
**Solvent:**	MeOH			3		369245	369826
**Container:**	1.5 eppendorf tube			**Mean**		**368913**	**369797**
**Conc. Tested:**	20 ug/mL			**SD**		**325**	**48**
**Fresh Lot ID:**	4.10.2020Stock_freeze-70C			**%CV**		**0.09**	**0.01**
**Stored Lot ID:**	04.10.2020 on bench overnight			**%Difference**			**0.24**
**RUN ID:**	UPLC_UV 4.11.2020			**n**		**3**	**3**

Stock Solution 1		Storage Time	Storage Temperature (^oC)	Replicate	Freshly Prepared Solution	OR Unexpired Stability Stock Solution	Stored Stability Stock Solution
					Peak Area		
		6 days	19-22	1		2080000	2110000
**Concentration:**	1mg/ml			2		2090000	2250000
**Solvent:**	MeOH			3		2090000	2050000
**Container:**	1.5 eppendorf tube			**Mean**		**2086667**	**2136667**
**Conc. Tested:**	25 ng/mL			**SD**		**5774**	**102632**
**Fresh Lot ID:**	4.10.2020Stock_freeze-70C			**%CV**		**0.28**	**4.8**
**Stored Lot ID:**	04.18.2020 on bench- 4.22.2020			**%Difference**			**2.4**
**RUN ID:**	7			**n**		**3**	**3**

Working Solution 1		Storage Time	Storage Temperature (^oC)	Replicate	Freshly Prepared Solution	OR Unexpired Stability Stock Solution	Stored Stability Stock Solution
					Peak Area		
		27 h	19-22	1		1810000	2100000
**Concentration:**	10 ug/mL			2		1950000	2070000
**Solvent:**	50% MeOH			3		1850000	1940000
**Container:**	1.5 eppendorf tube			**Mean**		**1870000**	**2036667**
**Conc. Tested:**	20 ng/mL			**SD**		**72111**	**85049**
**Fresh Lot ID:**	4.16.2020wk			**%CV**		**3.86**	**4.18**
**Stored Lot ID:**	5.21.2020 on bench overnight			**%Difference**			**8.9**
**RUN ID:**	14			**n**		**3**	**3**

Table 9D: BDCQ Solution Stability Continues

Working Solution 1		Storage Time	Storage Temperature (°C)	Replicate	Freshly Prepared Solution	OR Unexpired Stability Stock Solution	Stored Stability Stock Solution
					Peak Area		
		8 days	19-22	1	1250000	1400000	1290000
**Concentration:**	10 ug/mL			2	1280000	1320000	1240000
**Solvent:**	50% MeOH			3		1280000	1250000
**Container:**	1.5 eppendorf tube			**Mean**		**1333333**	**1260000**
**Conc. Tested:**	12.5 ng/mL			**SD**		**61101**	**26458**
**Fresh Lot ID:**	7.12.2020WK_QC			**%CV**		**4.58**	**2.10**
**Stored Lot ID:**	7.12.2020 WK on bench until 7.20.2020			**%Difference**			**-5.5**
**RUN ID:**	19			**n**		**3**	**3**

IS Working Solution		Storage Time	Storage Temperature (°C)	Replicate	frozen Solution (Control)	glass (1.5dr) 16x50mm	plastic (1.5mL Eppendorf tube)
					Peak Area		
		6 hr	21-25	1	28200	5110	26900
**Concentration:**	20 ng/mL			2	31200	3350	28900
**Solvent:**	50% MeOH			3	30900	3960	32800
**Container:**	Eppendorf tube			**Mean**	**30100**	**4140**	**29533**
**Conc. Tested:**	2 ng/mL			**SD**	**1652**	**894**	**3001**
**Fresh Lot ID:**	Apr 19, 2020 freeze			**%CV**	**5.5**	**21.6**	**10.2**
**Stored Lot ID:**	Apr 23, 2020 Wk on bench			**%Difference**		**-86**	**-1.9**
**RUN ID:**	10			**n**		**3**	**3**

IS Working Solution		Storage Time	Storage Temperature (°C)	Replicate	frozen Solution (Control)	glass (1.5dr) 16x50mm	plastic (1.5mL Eppendorf tube)
					Peak Area		
		23 hr	21-25	1	35900	5030	32300
**Concentration:**	20 ng/mL			2	37700	4490	36000
**Solvent:**	50% MeOH			3	36500	4490	32100
**Container:**	Eppendorf tube			**Mean**	**36700**	**4670**	**33467**
**Conc. Tested:**	2 ng/mL			**SD**	**917**	**312**	**2196**
**Fresh Lot ID:**	Apr 19, 2020 freeze			**%CV**	**2.5**	**6.7**	**6.6**
**Stored Lot ID:**	Apr 23, 2020 Wk on bench			**%Difference**		**-87**	**-8.8**
**RUN ID:**	10			**n**		**3**	**3**

Table 13A: AZM Hemolyzed samples

Hemolyzed samples Test QC Quantified off the Injected Curve					
		Run ID 9		Date/Time	4/24/2020
Replicates	Low TEST QC	High TEST QC	Statistics		
			[redacted]	Low TEST QC	High TEST QC
1	8.27	824	Nominal Conc.	6.00	800
2	6.45	832	Mean	7.34	845
3	7.29	880	SD	0.91	30
			%CV	12	3.6
[redacted]			%Dev from Nominal Conc.	22	5.7
			n	3	3
Control QC Quantified off the Injected Curve					
Replicates	Low TEST QC	High TEST QC		Low TEST QC	High TEST QC
1	6.66	817	Nominal Conc.	6.00	800
2	6.48	875	Mean	6.60	857
3	6.66	879	SD	0.10	35
			%CV	1.6	4.0
[redacted]			%Dev from Nominal Conc.	10	7.1
			n	3	3
% Difference between Treated (test QC) and Untreated (Control QC)				11	-1.4

Table 13B: HCQ Impact of hemolyzed samples

Hemolyzed samples Test QC Quantified off the Injected Curve					
		Run ID 9		Date/Time	4/24/2020
Replicates	Low TEST QC	High TEST QC	Statistics		
				Low TEST QC	High TEST QC
1	7.90	700	Nominal Conc.	6.00	800
2	7.55	788	Mean	7.59	753
3	7.31	772	SD	0.30	47
			%CV	3.9	6.2
[redacted]			%Dev from Nominal Conc.	26	-5.8
			n	3	3
Control QC Quantified off the Injected Curve					
Replicates	Low TEST QC	High TEST QC		Low TEST QC	High TEST QC
1	7.50	768	Nominal Conc.	6.00	800
2	7.06	712	Mean	7.36	746
3	7.53	759	SD	0.26	30
			%CV	3.6	4.0
[redacted]			%Dev from Nominal Conc.	23	-6.7
			n	3	3
(Control QC)				3.0	0.94

Table 13C: DHCQ Hemolyzed samples effect on quantitation

Hemolyzed samples Test QC Quantified off the Injected Curve					
		Run ID 9		Date/Time	4/24/2020
			Statistics		
Replicates	Low TEST QC	High TEST QC		Low TEST QC	High TEST QC
1	3.32	360	**Nominal Conc.**	**3.00**	**400**
2	3.12	333	**Mean**	3.17	350
3	3.06	356	**SD**	0.14	15
			**%CV**	4.3	4.2
[redacted]			**%Dev from Nominal Conc.**	5.6	-13
			**n**	3	3
**Control QC Quantified off the Injected Curve**					
Replicates	Low TEST QC	High TEST QC		Low TEST QC	High TEST QC
1	3.25	389	**Nominal Conc.**	**3.00**	**400**
2	2.90	369	**Mean**	2.98	381
3	2.79	384	**SD**	0.24	10
			**%CV**	8.1	2.7
[redacted]			**%Dev from Nominal Conc.**	-0.67	-4.8
			**n**	3	3
**% Difference between Treated (test QC) and Untreated (Control QC)**				6.3	-8.1

Table 13D: BDCQ Hemolyzed samples

Hemolyzed samples Test QC Quantified off the Injected Curve					
		Run ID 9		Date/Time	4/24/2020
			Statistics		
Replicates	Low TEST QC	High TEST QC		Low TEST QC	High TEST QC
1	1.54	179	**Nominal Conc.**	**1.50**	**200**
2	1.44	159	**Mean**	1.39	170
3	1.20	173	**SD**	0.17	10
			**%CV**	13	6.0
[redacted]			**%Dev from Nominal Conc.**	-7.1	-15
			**n**	3	3
**Control QC Quantified off the Injected Curve**					
Replicates	Low TEST QC	High TEST QC		Low TEST QC	High TEST QC
1	1.43	175	**Nominal Conc.**	**1.50**	**200**
2	1.41	170	**Mean**	1.46	176
3	1.53	182	**SD**	0.06	6
			**%CV**	4.4	3.4
[redacted]			**%Dev from Nominal Conc.**	-2.9	-12
			**n**	3	3
**(Control QC)**				-4.3	-3.0

Table 14A: AZM stability in blood at room teperature

Run ID = 9	5 min	22min	1 hr	2 hr	4 hr	6 hr
replicate 1	872	878	823	846	857	914
replicate 2	831	791	839	869	816	846
replicate 3	855	875	860	907	826	874
**Nominal Conc.**	800	800	800	800	800	800
**mean**	853	848	841	874	833	878
**SD**	21	47	19	31	21	34
**%CV**	2.4%	5.5%	2.2%	3.5%	2.6%	3.9%
**%dev**	6.6%	6.0%	5.1%	9.3%	4.1%	10%
**%difference (relative to 5 min)**		-0.55%	-1.4%	2.5%	-2.3%	3.0%

Table 14B: HCQ stability in blood at room teperature

Run ID = 9	5 min	22min	1 hr	2 hr	4 hr	6 hr
replicate 1	637	639	625	594	630	708
replicate 2	619	603	618	589	595	686
replicate 3	657	595	661	645	672	667
**Nominal Conc.**	800	800	800	800	800	800
**mean**	638	612	635	609	632	687
**SD**	19	96	23	31	39	21
**%CV**	3.0%	15.6%	3.6%	5.1%	6.1%	3.0%
**%dev**	-20%	-23%	-21%	-24%	-21%	-14%
**%difference (relative to 5 min)**		-4.0%	-0.47%	-4.4%	-0.84%	7.7%

Table 14C: DHCQ stability in blood at room teperature

Run ID = 9	5 min	22min	1 hr	2 hr	4 hr	6 hr
replicate 1	374	313	256	250	262	308
replicate 2	355	284	258	259	255	309
replicate 3	351	262	284	271	307	292
**Nominal Conc.**	400	400	400	400	400	400
**mean**	360	286	266	260	275	303
**SD**	12	61	16	11	28	10
**%CV**	3.4%	21%	5.9%	4.1%	10%	3.1%
**%dev**	-10%	-28%	-34%	-35%	-31%	-24%
**%difference (relative to 5 min)**		-20%	-26%	-28%	-24%	-16%
**%difference (relative to 22 min)**			-7.1%	-9.2%	-4.1%	5.8%

Table 14D: BDCQ stability in blood at room teperature

Run ID = 9	5 min	22min	1 hr	2 hr	4 hr	6 hr
replicate 1	127	132	123	121	133	141
replicate 2	129	126	115	130	122	141
replicate 3	135	114	133	137	150	122
**Nominal Conc.**	200	200	200	200	200	200
**mean**	130	124	124	129	135	135
**SD**	4	39	9	8	14	11
**%CV**	3.2%	31%	7.3%	6.2%	10.4%	8.1%
**%dev**	-35%	-38%	-38%	-35%	-33%	-33%
**%difference (relative to 5 min)**		-4.9%	-5.1%	-0.77%	3.6%	3.3%

Table 15A. Effect of K2EDTA on AZM quantitation

Run ID = 13	Plasma Lot#	QC	Nominal Conc. (ng/mL)	Calculated conc, ng/mL			mean, ng/mL	STD, ng/mL	%CV	%dev from control
				1	2	3				
K3EDTA (control)	LS 24 09280	low	6.00	5.64	5.57	6.09	5.77	0.28	4.9	
		Med	60.0	58.7	65.4	57.4	60.5	4.3	7.1	
		high	800	834	918	866	873	42	4.9	
K2EDTA	HMN392707	low	6.00	5.68	5.83	5.52	5.68	0.16	2.7	-1.6%
		high	800	802	841	765	803	38	4.7	-8.0%
	HMN392708	low	6.00	5.47	5.81	5.29	5.52	0.26	4.8	-4.2%
		high	800	813	846	828	829	17	2.0	-5.0%

Table 15B. Effect of K2EDTA on HCQ quantitation

Run ID = 13	Plasma Lot#	QC	Nominal Conc. (ng/mL)	Calculated conc, ng/mL			mean, ng/mL	STD, ng/mL	%CV	%dev from control
				1	2	3				
K3EDTA (control)	LS 24 09280	low	6.00	5.25	6.40	5.66	5.77	0.58	10	
		Med	60.0	65.1	66.2	59.4	63.6	3.65	5.7	
		high	800	814	842	807	821	18.52	2.3	
K2EDTA	HMN392707	low	6.00	6.17	5.95	5.98	6.03	0.12	2.0	4.6%
		high	800	822	810	675	769	81.63	11	-6.3%
	HMN392708	low	6.00	6.30	6.97	5.30	6.19	0.84	14	7.3%
		high	800	872	812	831	838	30.66	3.7	2.1%

Table 15C. Effect of K2EDTA on DHCQ quantitation

Run ID = 13	Plasma Lot#	QC	Nominal Conc. (ng/mL)	Calculated conc, ng/mL			mean, ng/mL	STD, ng/mL	%CV	%dev from control
				1	2	3				
K3EDTA (control)	LS 24 09280	low	3.00	2.68	2.89	3.31	2.96	0.32	11	
		Med	30.0	31.0	32.1	30.1	31.1	1.0	3.2	
		high	400	410	430	424	421	10	2.4	
K2EDTA	HMN392707	low	3.00	3.06	2.61	2.86	2.84	0.23	7.9	-3.9%
		high	400	396	372	338	369	29	7.9	-13%
	HMN392708	low	3.00	3.16	3.55	2.98	3.23	0.29	9.0	9.1%
		high	400	414	419	432	422	9	2.2	0.1%

Table 15D. Effect of K2EDTA on BDCQ quantitation

Run ID = 13	Plasma Lot#	QC	Nominal Conc. (ng/mL)	Calculated conc, ng/mL			mean, ng/mL	STD, ng/mL	%CV	%dev from control
				1	2	3				
K3EDTA (control)	LS 24 09280	low	1.50	1.30	1.64	1.54	1.49	0.17	12	
		Med	15.0	14.8	15.3	14.5	14.9	0.4	2.7	
		high	200	197	226	211	211	15	6.9	
K2EDTA	HMN392707	low	1.50	1.28	1.57	1.49	1.45	0.15	10	-3.1%
		high	200	209	199	156	188	28	15	-11%
	HMN392708	low	1.50	1.49	1.69	1.79	1.66	0.15	9.2	11%
		high	200	206	211	212	210	3	1.5	-0.8%
