# Supplementary material for: Development and validation of an LC-MS/MS method for determination of hydroxychloroquine, its two metabolites, and azithromycin in EDTA-treated human plasma
Source: PLoS One. 2021 Mar 5;16(3):e0247356. doi: 10.1371/journal.pone.0247356 (PMC7935301; doi:10.1371/journal.pone.0247356)
Supplement: S1 File — (PDF) [file pone.0247356.s005.pdf]

Determination of azithromycin, hydroxychloroquine, desethylhydroxychloroquine, and bisdesethylchloroquine in K_3EDTA-human plasma by LC-MS/MS

Standard Operating Procedure

Version 1.3

August 25, 2020

De-identified

Introduction

Azithromycin (AZM) is a 2nd generation macrolide antibacterial, inhibiting bacterial protein synthesis[1], it also shows moderate activity against malaria and used in combination with chloroquine for chemoprevention of malaria[2]. Its C_{max} has been reported to be around 400 ng/mL following a 500 mg single dose[3], and higher concentrations are expected following multiple doses. AZM has low oral bioavailability (17-37%) and low plasma protein binding (~30%) but long elimination half life (~70hr)[1]. It is accumulated in tissues and blood leukocytes. Recently it was also used in combination with hydroxychloroquine (HCQ) to treat an emerging coronavirus COVID-19[4].

HCQ was initially developed as an antimalarial drug, but also used for autoimmune diseases such as rheumatoid Arthritis for several decades[5,6]. Recent studies found it was active for COVID-19[7] and used to treat patients[4]. HCQ is 50% bound to plasma proteins, absorbed completely and rapidly (70-80% in gastrointestinal tract)[8]. It has a very long half-life (up to 40days), the C_{max} after multiple doses may reach up to 1000 ng/mL[9,10]. In liver, HCQ is metabolized to desethyl-chloroquine (DCQ), desethyl-hydroxychloroquine (DHCQ) which is active, and bis-desethyl-hydroxychloroquine (BDCQ) which has been implicated in toxicity[11].

In an emergency response to the outbreak of COVID-19, our lab developed and validated an LCMS-MS method to assess the pharmacokinetics of AZM, HCQ, DHCQ, and BDCQ in patients' plasma in order to support clinical trials to combat the spread of this pandemic. The assay uses deuterated compounds as the internal standards (IS). The calibration curve concentration range for the assay is 2-1000 ng/mL for AZM and HCQ, 1-500 ng/mL for DHCQ, and 0.5-250 ng/mL for BDCQ, with only 20 µL plasma volume.

Principle of the Method

AZM, HCQ, DHCQ, and BDCQ along with their deuterated internal standards (AZM-d_5, HCQ-d_4, DHCQ-d_4, BDCQ-d_4) are extracted from EDTA plasma by solid-phase extraction with HLB micro-elution plate. The processed sample is injected onto a PFP column (2.0 $\times$ 50 mm, 3 µm) eluted with water and acetonitrile, each with 0.05% TFA, in a gradient mode. ESI in positive mode and multiple reaction monitoring (MRM) are used, and ion pairs m/z 749.6$\rightarrow$591.6 for AZM, 336.1$\rightarrow$247.1 for HCQ, 308.1$\rightarrow$179.1 for DHCQ, and 264.1$\rightarrow$179.1 for BDCQ are selected for quantification, The ion pairs for the corresponding ISs are 754.6$\rightarrow$596.6, 342.1$\rightarrow$253.1, 314.1$\rightarrow$181.1, and 270.1$\rightarrow$181.1. HCQ-d_4, DHCQ-d_4, and BDCQ-d_4 utilized the ions from the less abundant chlorine isotope (^{37}Cl) to overcome the interference from naturally occurring isotopes of HCQ, DHCQ, and BDCQ, respectively. The retention times are 0.78 min for BDCQ, 0.79 min for DHCQ, 0.92 min for HCQ and 1.87min for AZM, respectively. Total run time is 3.5 min per sample. The LC-MS/MS system is operated at room temperature (25 ± 5 $^{\circ}$C controlled by an air conditioner). Calibration curve standards and quality controls are prepared in blank EDTA human plasma from separately weighted and prepared stock solutions. Quadratic least square regression with a $1/x^2$ weighting factor is applied to AZM calibration curve and linear least square regression is applied to calibration curve with a

weighting factor of 1/x for HCQ, DHCQ and BDCQ. Quantification utilizes the peak area ratio of analytes to the corresponding IS.

Analytes and Internal Standards

Azithromycin $C_{38}H_{72}N_2O_{12}\cdot 2H_2O$ MW: 785
CAS# 83905-01-5
Free base form: $C_{38}H_{72}N_2O_{12}$ MW: 749 EM: 748.51
USP reference standard. Sigma-Aldrich Inc Cat# 1046056.
Synonyms: (2R,3S,4R,5R,8R,10R,11R,12S,13S,14R)-11-[(2S,3R,4S,6R)-4-(dimethylamino)-3-hydroxy-6-methyloxan-2-yl]oxy-2-ethyl-3,4,10-trihydroxy-13-[(2R,4R,5S,6S)-5-hydroxy-4-methoxy-4,6-dimethyloxan-2-yl]oxy-3,5,6,8,10,12,14-heptamethyl-1-oxa-6-azacyclopentadecan-15-one

Azithromycin-d$_5$ $C_{38}H_{67}D_5N_2O_{12}$ MW: 754.02 EM: 753.55
CAS# NA
TRC. Cat#A927004
Synonyms: Sumamed-d5; Trozocina-d5; Zithromaz-d5; Zitromax-d5.

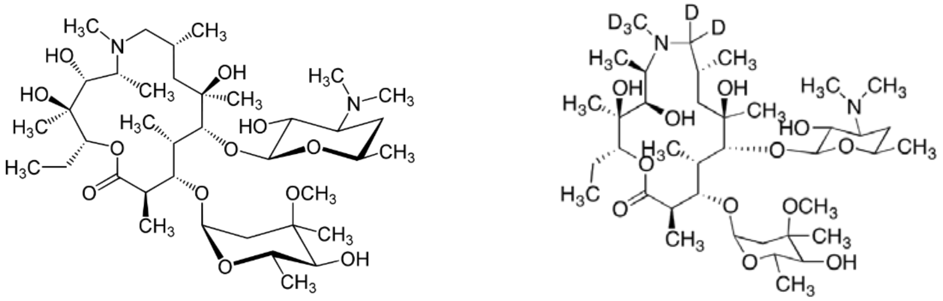

Azithromycin Azithromycin-d5

Figure 1A. chemical structures of Azithromycin and its internal standard.

Hydroxychloroquine sulfate $C_{18}H_{28}ClN_3O_5S$ MW: 433.95
CAS# 118-42-3
Free base form: $C_{18}H_{26}ClN_3O$ MW: 335.9 EM: 335.18
AK Scientific, Inc. Cat# J10260
Sigma-Aldrich USP reference, Cat# 1327000.
Synonyms: 2-[4-[(7-chloroquinolin-4-yl)amino]pentyl-ethylamino]ethanol]; Plaquenil, oxychloroquine, WIN1258.

Hydroxychloroquine-d4 sulfate $C_{18}H_{24}D_4ClN_3O_5S$ MW: 437.97
CAS# 1216432-56-2
Free base form: $C_{18}H_{22}D_4ClN_3O$ MW 339.90 EM: 339.20
Santa Cruz Biotech. Cat#SC-280804.
Synonyms: 2-[[4-[(7-chloroquinolin-4-yl)amino]-1,1,2,2-tetradeuteriopentyl]-ethylamino]ethanol

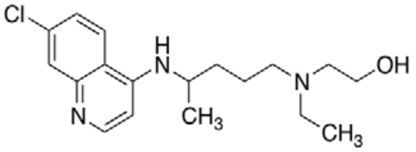
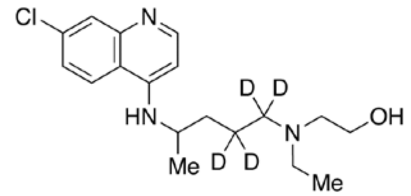

Hydroxychloroquine Hydroxychloroquine-d_4

Figure 1B. Chemical structures of hydroxychloroquine and its internal standard.

Desethyl hydroxychloroquine $C_{16}H_{22}ClN_3O$ MW: 307.82 EM: 307.15
CAS# 4298-15-1
TRC. Cat# C573505
Synonyms: Cletoquine; 2-[4-[(7-chloroquinolin-4-yl)amino]pentylamino]ethanol

Desethyl hydroxychloroquine-d4 $C_{16}H_{18}D_4ClN_3O$ MW: 311.84 amu EM: 311.17
CAS# 1216461-57-2
TRC. Cat# C573505.
Synonyms: Chloroquinolin-4-yl)amino]-1,1,2,2-tetradeuteriopentyl]amino]ethanol

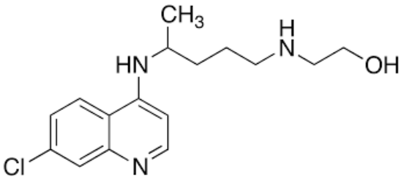
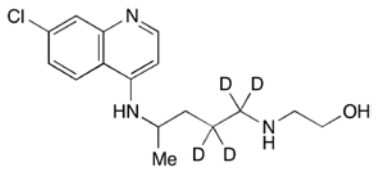

Desethyl hydroxychloroquine Desethyl hydroxychloroquine-d4

Figure 1C. Chemical structures of desethylhydroxycloroqine and its internal standard.

Bisdesethyl chloroquine $C_{14}H_{18}ClN_3$ MW: 263.77 EM: 263.12
CAS# 4298-14-0
TRC. Cat# D440960.
Synonyms: 4-*N*-(7-chloroquinolin-4-yl)pentane-1,4-diamine

Bisdesethyl chloroquine-d4 $C_{14}H_{14}D_4ClN_3$ MW: 267.79 EM: 267.14
CAS# 1215797-41-3
TRC. Cat# D440962.
Synonyms: 4-[(4-Amino-1-methylbutyl)amino]-7-chloroquinoline-d4

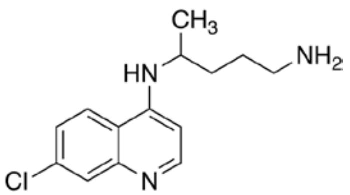
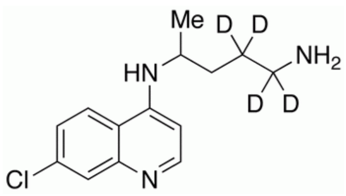

Bisdesethyl chloroquine Bisdesethyl chloroquine-d4

Figure 1D. Chemical structures of bisdesethyl chloroquine and its internal standard.

Specimen Handling

Whole blood from specimens should be collected in a 4mL K_3EDTA or K_2EDTA plastic container, and inverted gently 8-10 times. Within 4 hours of collection, specimen samples should be centrifuged at 2000xg for 10 minutes as centrifuge force less than 1200g caused variation on plasma HCQ concentration [12]. The resulting plasma samples are transferred into plastic cryogenic tubes and placed at -70°C for long term storage. If shipping of samples is needed, samples should be kept frozen and shipped on dry ice.

Although hemolysis does not impact the method performance, it should be avoided due to higher drug concentrations in blood cells than those in plasma.

Equipment

1) Sciex API5000 coupled with Shimadzu Prominence 20AD^{XR} UFLC pumps and SIL-20AC^{XR} autosampler, managed with the software Analyst® 1.6.2. (Cat#NA)
2) Bench-top Eppendorf centrifuge 5417C (Cat#NA)
3) Finnpipette 100 -1000 µl (Fisher Scientific Cat# 21-377-821), 20 – 200 µl (Fisher Scientific Cat# 21-377-820), 10-100 µL (Fisher Scientific Cat# 21-377-819), 5-40 µl (Fisher Scientific Cat# not available)
4) Eppendorf reference 2 – 20 µl (Fisher Scientific Cat# S304663), 0.5 – 10µL (Fisher Scientific, Eppendorf Cat# 2231000604)
5) Eppendorf repeater plus pipettor (Fisher Scientific Cat# 21-380-9)
6) Thermolyne MaxiMix Plus Vortex Mixer (Fisher Scientific Cat# 12-815-18)
7) Tube Rotator (Stuart® Rotator SB3, Bibby Scientific Ltd Cat# 11496548)
8) Mettler Toledo PB153-S balance (Fisher Scientific Cat# 01-916-256)
9) Mettler Toledo XS105 dual range balance (Fisher Scientific Cat# 01-910-12).
10) Mettler Toledo MP220 PH meter (Cat# 01915102)
11) Sonicator (Fisher Scientific, model# FS-14).
12) CaptiVac™ Vacuum Collar (Varian Inc., Walnut Creek, CA, Cat# A796)

Chemicals

Caution: consult SDS of chemicals below prior to handling them

1) Acetonitrile Optima® LC/MS Grade (CH_3CN) (Fisher Scientific Cat# A9554) (toxic)
2) Methanol Optima® LC/MS Grade (MeOH) (Fisher Scientific Cat# A4564) (toxic)
3) HPLC Water Optima® LC/MS Grade (Fisher Scientific Cat# W64)
4) Trifluoroacetic acid Optima® LC/MS Grade (Fisher Scientific Cat# A116-50)
5) Formic acid Optima® LC/MS Grade (HCOOH) (Fisher Scientific Cat# A117-50)
6) Sodium hydroxide (LabChem, Fisher Scientific Cat# LC239001)

7) 2-propanol (J.T. Bakercat#933403, Fisher Scientific Cat# 14-650-220)

Consumables

1) Eppendorf 1.5 mL snap-top micro-centrifuge tubes (Fisher Scientific Cat# 02-681-239)
2) Fisherbrand 50 mL centrifuge tubes (Fisher Scientific Cat# 06-443-18)
3) Waters 1mL round bottom 96-well plate (Waters Cat# 186002481) and cap mat (part# 186002483).
4) HLB μ-elution plate (part#186001828BA, 2mg sorbent per well, 30 μm particle size)
5) Pipettor tips, (Fisher Scientific Cat# 02-707-108, 02-707-101, and 21-197-2F)
6) Human plasma, EDTA (Biological Specialty Corp Cat# 130-21)
7) Pursuit PFP column (50 × 2.0 mm, 3 μm, Agilent Tech, Cat# A3051050X020) and guard column (10× 2.0 mm, 3 μm, Agilent Tech, Cat# A3051MG2)
8) Eppendorf Combitips plus pipet tips (Fischer Scientific Cat# 21-381-329)

Preparation of Reagents

Volumes and weights may be scaled up and down as needed.

Special note: TFA is extremely caustic, and should be handled with extreme care using glass volumetric pipet..

Mobile phase A (0.05%TFA): Add 0.5mL TFA into 1L water, cap the bottle, invert the bottle 5-10 times to mix, then degas with sonication under vacuum. Stored at room temperature and expire after 60 days.

Mobile phase B (0.05%TFA in acetonitrile): Add 0.5mL TFA into 1 L acetonitrile, cap the bottle, invert the bottle 5-10 times to mix, and degas with sonication under vacuum. Stored at room temperature and expire after 60 days.

Needle wash solution (85% MeCN 0.1%FA): Add 150 mL water into 850 mL acetonitrile, add 1mL formic acid, cap the bottle, invert the bottle 5-10 times to mix and degas under vacuum with sonication. Expire after 60 days.

MeOH-water (1:9, v/v) or 10% MeOH: Add 5mL MeOH into 45mL water. Vortex-mixed at 2000 rpm for 5-10 sec. Store at room temperature. Expire after 3 months.

MeOH-water (50:50, v/v) or 50% MeOH: Add 25mL water into 25 mL MeOH. Vortex-mixed at 2000 rpm for 5-10 sec. Store at room temperature. Expire after 3 months.

MeOH with 0.5% FA: To 50mL MeOH is added 0.250mL formic acid (FA). Vortex-mixed at 2000 rpm for 5-10 sec. Store at room temperature. Expire after 3 months.

0.1N sodium hydroxide: dissolve 0.4 g sodium hydroxide (s) in 100 mL water. Store at room temperature. Expire after 3 months.

80% Isopropanol (column wash): add 200 mL water to 800 mL IPA, cap the bottle, invert the bottle 5-10 times to mix. Store at room temperature. Expire after 60 days.

Preparation of Standard Curve and Quality Controls

Note: Due to adsorption of drugs on glass surface, all stock and working solutions should be prepared in plastic containers/tubes.

Calibration Standard Stock Solutions

Stock and working solutions for HCQ are made in water; For other three analytes (AZM, DHCQ and BDCQ), stock solutions are made in MeOH and working solutions are made in 50% MeOH. Volumes and weights may be scaled up and down as needed.

A) AZM primary stock solution (2.00 mg/mL)

Weigh ~2 mg AZM (USP reference) and dissolve with 50% MeOH in a 1.5mL Eppendorf tube to obtain a final concentration of 2.00 mg/mL AZM. Vortex-mix for at least 5 sec at 2000 rpm and then rotate on a mixer for at least 30 minutes at a minimum speed of 30 rotations per minute. Seal with parafilm and store at -70 °C. Stock solution in 50% MeOH is stable at -70 °C for at least 45 days and at room temperature for at least 23hr. Further stability is under investigation.

B) HCQ primary stock solution (2.00 mg/mL)

Weigh ~2 mg HCQ (hydroxychloroquine sulfate, conversion factor is 0.774) and dissolve with water in a 1.5mL Eppendorf tube to obtain a final concentration of 2.00 mg/mL HCQ in base form. Vortex for no less than 5 sec at 2,000 rpm and then rotate on mixer for at least 30 minutes at a minimum speed of 30 rotations per minute. Seal with parafilm and store at -70 °C. It is stable for at least 63 days at -70 °C and 5 days at room temperature. Further stability is under investigation.

C) DHCQ primary stock solution (1.00 mg/mL)

Weigh ~1 mg DHCQ (desethyl hydroxychloroquine, base form) and dissolve with MeOH in a 1.5 mL Eppendorf tube to obtain a final concentration of 1.00 mg/mL DHCQ in base form. Vortex for no less than 5 sec at 2,000 rpm and then rotate on mixer for at least 30 minutes at a minimum speed of 30 rotations per minute. Seal with parafilm and store at -70 °C. Stock solution is stable at -70°C for at least 1 day and at room temperature for at least 14 hr. Further stability is under investigation.

D) BDCQ primary stock solution (1.00 mg/mL)

Weigh ~1 mg BDCQ (bisdesethylhydroxychloroquine, free base form) and dissolve with MeOH in a 1.5 mL Eppendorf tube to obtain a final concentration of 1.00 mg/mL BDCQ in base form. Vortex for no less than 5 sec at 2,000 rpm and then rotate on mixer for at least 30 minutes at a minimum speed of 30 rotations per minute. Seal with parafilm and store at -70 °C. Stock solution is stable at -70 °C for at least 6 days and at room temperature for at least 6 days. Further stability is under investigation.

Calibration Standard Working Solutions (AZM/HCQ/DHCQ/BDCQ 40/40/20/10 μg/mL)

Pipet 20 μL AZM (2.00 mg/mL), 20 μL HCQ (2.00 mg/mL), 20 μL DHCQ (1 mg/mL), and 10 μL BDCQ (1 mg/mL) in 930 μL 50% MeOH in a 1.5 mL Eppendorf tube and vortex-mix at 2000 rpm for at least 10 sec. Seal with parafilm and store at -70 °C. AZM (40 μg/mL) is stable at -70 °C for at least 36 days and room temperature overnight. HCQ (40 μg/mL in water) is stable at -70 °C for at least 63 days and room temperature overnight. The mixed working solution of the 4 analytes in 50% MeOH is stable at room temperature for at least 8 days. Further stability is under investigation.

Alternatively, a combined working solution for AZM, DHCQ and BDCQ may be prepared as follows: Pipet 20 μL AZM (2.00 mg/mL), 20 μL DHCQ (1 mg/mL), and 10 μL BDCQ (1 mg/mL) in 950 μL 50% MeOH in a 1.5 mL Eppendorf tube and vortex-mix at 2000 rpm for at least 10 sec to give combined working solution (AZM/DHCQ/BDCQ 40/20/10 μg/mL). HCQ working solution (40 μg/mL) was prepared by pipetting 20 μL HCQ (2.00 mg/mL) into 980 μL water in a 1.5 mL Eppendorf tube and vortex-mixing at 2000 rpm for at least 10 sec.

Calibration Standards in plasma

Calibration standards are prepared in blank pooled K_3EDTA human plasma in 1.5 mL plastic Eppendorf tubes. Combined working solution is used and the solutions spiked into blank plasma should be ≤5% to minimize alteration of matrix. Pipet 25 μL of a combined standard working solution (AZM/HCQ/DHCQ/BDCQ 40/40/20/10 μg/mL in 50% MeOH) into 975 uL blank plasma in a 1.5 mL plastic Eppendorf tubes and vortex-mix for at least 10 sec at 2000 rpm, giving concentrations at 1000 ng/mL for AZM and HCQ, 500 ng/mL for DHCQ, and 250 ng/mL for BDCQ (plasma standard #9).

Alternatively, pipet 25 μL of a combined working solution (AZM/DHCQ/BDCQ 40 /20/10 μg/mL in 50% MeOH) and 25 μL HCQ working solution (40 μg/mL in water) into 950 μL blank plasma in a 1.5 mL plastic Eppendorf tubes and vortex-mix for at least 10 sec at 2000 rpm to give plasma standard #9.

Other calibration standards are prepared by serial dilutions as described in table 1 at the concentrations of 2/2/1/0/5, 5/5/2.5/1.25, 10/10/5/2.5, 20/20/10/5, 50/50/25/12.5, 100/100/50/25, 200/200/100/50, and 500/500/250/125 ng/mL

for AZM/HCQ/DHCQ and BDCQ, respectively. Vortex mix for at least 10 sec at 2000 rpm.

It is recommended to prepare calibrators freshly on the day to be used for analysis. Alternatively frozen calibrators within the validated stability period may be used. Plasma samples are stable at – 70 °C for at least 38 days and 4 freeze-thaw cycles.

Table 1. Preparation of calibration standards

Std#	AZM/HCQ/DHCQ/BDCQ work solution or Std, ng/mL	Vol of sol, μL	Blank Plasma Vol, μL	Plasma std final AZM/HCQ/DHCQ/ BDCQ conc, ng/mL
9	40,000/40,000/20,000/ 10,000	25.0	975	1000/1000/500/ 250
8	Std#9	100	100	500/500/250/125
7	Std#9	50.0	200	200/200/100/50
6	Std#9	20.00	180	100/100/50/25
5	Std#8	20.00	180	50/50/25/12.5
4	Std#7	20.00	180	20/20/10/5
3	Std#6	20.00	180	10/10/5/2.5
2	Std#5	20.00	180	5/5/2.5/1.25
1	Std#4	20.00	180	2/2/1/0.5

<u>Validation (Quality Control) Stock Solutions</u>

Quality control (QC) stock solution is prepared from a separate weighing than that used for making calibration standards. Source and drug form (free base or salt) can be identical to or different from that used for the calibration standard. Volumes and weights may be scaled up and down as needed. The differences of the QC stocks from the stocks used for calibrators should be <10%, preferably <5%.

Alternatively, QC stock solutions may be the same as calibrator standard stocks only if the accuracy of stocks is verified, either from a certified stock solution or by a separately weighed stock.

A) AZM QC primary stock solution (2.00 mg/mL)

Weigh ~2 mg AZM (USP reference) and dissolve with MeOH or MeOH-water (1:1, v/v) in a 1.5 mL Eppendorf tube to obtain a final concentration of 2.00 mg/mL AZM. Vortex for no less than 5 sec at 2,000 rpm and then rotate on mixer for at least 30 minutes at a minimum speed of 30 rotations per minute. Seal with parafilm and store at -70 °C. Stock solution in MeOH-water (1:1, v/v) is stable at -70 °C for at least 45 days and at room temperature for at least 23hr. Further

stability is under investigation. Stock solution in MeOH is stable at room temperature for at least 14 hr (6 days for stock at 0.5mg/mL).

B) HCQ QC primary stock solution (2.00 mg/mL)

Weigh ~2 mg HCQ (hydroxychloroquine sulfate, conversion factor is 0.774) and dissolve with water in a 1.5mL Eppendorf tube to obtain a final concentration of 2.00 mg/mL HCQ in base form. Vortex for no less than 5 sec at 2,000 rpm and then rotate on mixer for at least 30 minutes at a minimum speed of 30 rotations per minute. Seal with parafilm and store at -70 °C. It is stable for at least 63 days at -70 °C and 5 days at room temperature. Test for longer term stability is pending. Expiration date is under investigation.

C) DHCQ QC primary stock solution (1.00 mg/mL)

Weigh ~1 mg DHCQ (desethyl hydroxychloroquine, base form) and dissolve with MeOH in a 1.5 mL Eppendorf tube to obtain a final concentration of 1.00 mg/mL DHCQ in base form. Vortex for no less than 5 sec at 2,000 rpm and then rotate on mixer for at least 30 minutes at a minimum speed of 30 rotations per minute. Seal with parafilm and store at -70 °C. Stock solution is stable at -70 °C for at least 1 day and at room temperature for at least 14 hr. Further stability is under investigation.

D) BDCQ QC primary stock solution (1.00 mg/mL)

Weigh ~1 mg BDCQ (bisdesethylhydroxychloroquine, free base form) and dissolve with MeOH in a 1.5 mL Eppendorf tube to obtain a final concentration of 1.00 mg/mL BDCQ in base form. Vortex for no less than 5 sec at 2,000 rpm and then rotate on mixer for at least 30 minutes at a minimum speed of 30 rotations per minute. Seal with parafilm and store at -70 °C. Stock solution is stable at -70 °C for at least 6 days and at room temperature for at least 6 days. Further stability is under investigation.

Due to the limited source and high cost of DHCQ and BDCQ standards, the stock solutions of DHCQ and BDCQ may be alternatively made with pre-weighed drug standards from the vendor, the exact weight from the vendor should be accurate with at least 2 digits after decimal point.

<u>QC Working Solutions</u>

1) QC working solution 1(40/40/20/10 μg/mL AZM/HCQ/DHCQ/BDCQ)

A combined working solution may be prepared as follows: Pipet 20 μL AZM (2.00 mg/mL), 20 μL HCQ (2.00 mg/mL), 20 μL DHCQ (1 mg/mL), and 10 μL BDCQ (1 mg/mL) in 930 μL 50% MeOH in a 1.5 mL Eppendorf tube and vortex-mix at 2000 rpm for at least 10 sec. AZM (40 μg/mL) is stable at -70 °C for at least 36 days and room temperature overnight. The mixed working solution of the 4 analytes in 50% MeOH is stable at room temperature for at least 8 days. Further stability test is pending.

Alternatively, a 3-analytes mixed working solution (40/20/10μg/mL AZM/DHCQ/BDCQ) may be prepared in 50% MeOH by spiking 20 μL AZM (2.00 mg/mL), 20 μL DHCQ (1 mg/mL), and 10 μL BDCQ (1 mg/mL) in 950 μL 50% MeOH in a 1.5 mL Eppendorf tube and vortex-mix at 2000 rpm for at least 10 sec. HCQ working solution (40 μg/mL) may be prepared in water by spiking 20 μL HCQ (2.00 mg/mL) in 980 μL water in a 1.5 mL Eppendorf tube and vortex-mix at 2000 rpm for at least 10 sec. HCQ working solution (40 μg/mL in water) is stable at -70 °C for at least 2 months and room temperature overnight. Further stability test is pending.

2) QC working solution 2(3/3/1.5/0.75 μg/mL AZM/HCQ/DHCQ/BDCQ)

Pipet 30 μL QC working solution 1 (40/40/20/10 μg/mL AZM/HCQ/DHCQ/BDCQ)in 370 μL 50% MeOH in a 1.5 mL Eppendorf tube and vortex-mix at 2000 rpm for at least 10 sec. Prepared freshly.

3) QC working solution 3(300/300/150/75 ng/mL AZM/HCQ/DHCQ/BDCQ)

Pipet 40 μL QC working solution 2 (3/3/1.5/0.75 μg/mL AZM/HCQ/DHCQ/BDCQ)in 360 μL 50% MeOH in a 1.5 mL Eppendorf tube and vortex-mix at 2000 rpm for at least 10 sec. Prepared freshly.

QC Plasma samples

QC Low (6.00/6.00/3.00/1.50 ng/mL for AZM/HCQ/DHCQ/BDCQ)

Add 20.0μL of QC working solution 3 (300/300/150/75 ng/mL AZM/HCQ/DHCQ/BDCQ)into 0.980 mL blank human plasma (K_3EDTA or K_2EDTA as the anticoagulant) in a 1.5 mL Eppendorf tube to make the final concentration of 6.00/6.00/3.00/1.50 ng/mL for AZM/HCQ/DHCQ/BDCQ. Rotate on mixer for around 30 minutes at a speed of at least 30 rpm. Pipet 85μL aliquots into labeled 1.5 mL Eppendorf tubes and keep frozen until analyzed. Storage -70°C. Plasma samples are stable at – 70 °C for at least 38 days and 4 freeze-thaw cycles. Longer storage time is under investigation.

QC Medium (60.0/60.0/30.0/15.0 ng/mL for AZM/HCQ/DHCQ/BDCQ)

Add 20.0μL of QC working solution 2 (3/3/1.5/0.75 μg/mL AZM/HCQ/DHCQ/BDCQ) into 0.980 mL blank human plasma (K_3EDTA or K_2EDTA as the anticoagulant) in a 1.5 mL Eppendorf tube to make the final concentration of 60.0/60.0/30.0/15.0 ng/mL for AZM/HCQ/DHCQ/BDCQ. Rotate on mixer for around 30 minutes at a speed of at least 30 rpm. Pipet 85μL aliquots into labeled 1.5 mL Eppendorf tubes and keep frozen until analyzed. Storage -70°C. Plasma samples are stable at – 70 °C for at least 38 days and 4 freeze-thaw cycles. Longer storage time is under investigation.

QC High (800/800/400/200 ng/mL for AZM/HCQ/DHCQ/BDCQ)

Add 20.0 μL of QC working solution 1 (40/40/20/10 μg/mL AZM/HCQ/DHCQ/BDCQ) into 0.980 mL blank human plasma (K_3EDTA or K_2EDTA

as the anticoagulant) in a 1.5 mL Eppendorf tube to make the final concentration of 800/800/400/200 ng/mL for AZM/HCQ/DHCQ/BDCQ. Mix on a rotor at ≥ 30 rpm for around 30 minutes. Pipet 85 µL aliquots into labeled 1.5 mL Eppendorf tubes and keep frozen at -70°C until analyzed. Plasma samples are stable at – 70 °C for at least 38 days and 4 freeze-thaw cycles. Longer storage time is under investigation.

Alternatively, QC medium and low samples may also be prepared by serial dilution: QC high (30 µL) is added into 370 µL blank plasma and vortex-mix at 2000 rpm for at least 10 seconds to give QC medium. QC medium (40 µL) is added into 360 µL blank plasma and vortex-mix at 2000 rpm for at least 10 seconds to give QC low.

Preparation of Internal Standard Solution
Volumes and weights may be scaled up and down as needed.

Internal Standard Primary Stock Solution (1.0 mg/mL)

A) AZM-d_5 (1 mg/mL): Add 1.000 mL MeOH to a vial containing 1.0 mg AZM-d_5 to obtain a final concentration of 1.0 mg/mL. Vortex at 2000 rpm for at least 10 sec, then mix on a rotor at ≥30 rpm for around 30 minutes. Storage -70 °C. Expiration date is under investigation.

B) HCQ-d_4 (1 mg/mL): Add 1.000 mL water to a vial containing 1.0 mg HCQ-d_4 free base to obtain a final concentration of 1.0 mg/mL. Vortex 1 min, then mix on a rotor at ≥30 rpm for around 30 minutes. Storage -70 °C. Expiration date is under investigation.

C) DHCQ-d_4 (1 mg/mL): Add 1.000 mL MeOH to a vial containing 1.0 mg DHCQ-d_4 free base to obtain a final concentration of 1.0 mg/mL. Vortex at 2000 rpm for at least 10 sec, then mix on a rotor at ≥30 rpm for around 30 minutes. Storage -70 °C. Expiration date is under investigation.

D) BDCQ-d_4 (1 mg/mL): Add 1.000 mL MeOH to a vial containing 1.0 mg BDCQ-d_4 free base to obtain a final concentration of 1.0 mg/mL. Vortex at 2000 rpm for at least 10 sec, then mix on a rotor at ≥30 rpm for around 30 minutes. Storage -70 °C. Expiration date is under investigation.

Internal Standard Secondary Stock Solution (10 µg/mL)

A) AZM-d_5 (10 µg/mL): Pipet and mix 10.00 µL of the primary stock solution (1.0 mg/mL) with 0.990 mL 50% MeOH in a 1.5 mL Eppendorf tube to make the final concentration of 10 µg/mL. Vortex at 2000 rpm for at least 10 seconds. Storage -70 °C. Expiration date is under investigation.

B) HCQ-d_4 (10 µg/mL): Pipet and mix 10.00 µL of the primary stock solution (1.0 mg/mL) with 0.990 mL water in a 1.5 mL Eppendorf tube to make the final concentration of 20 µg/mL. Vortex at 2000 rpm for at least 10 seconds. Storage -70 °C. Expiration date is under investigation.

C) DHCQ-d_4 (10 µg/mL): Pipet and mix 10.00 µL of the primary stock solution (1.0 mg/mL) with 0.990 mL 50% MeOH in a 1.5 mL Eppendorf tube to make the final concentration of 10 µg/mL. Vortex at 2000 rpm for at least 10 seconds. Storage -70 °C. Expiration date is under investigation.

D) BDCQ-d_4 (10 µg/mL): Pipet and mix 10.00 µL of the primary stock solution (1.0 mg/mL) with 0.990 mL 50% MeOH in a 1.5 mL Eppendorf tube to make the final concentration of 10 µg/mL. Vortex at 2000 rpm for at least 10 seconds. Storage -70 °C. Expiration date is under investigation.

<u>Internal Standard Working Solution</u> (100/40/20/20 ng/mL for AZM-d_5/HCQ-d_4 /DHCQ-d_4/BDCQ-d_4)

From the secondary stock solutions (10 µg/mL), add 200 µL of AZM-d_5, 80 µL of HCQ-d_4, 40 µL of DHCQ-d_4 and 40 µL of BDCQ-d_4 into 19.64 mL 50%MeOH in a plastic centrifuge tube and mixed well. Vortex at 2000 rpm for at least 10 seconds. Storage -70 °C. The combined IS solution is stable at room temperature for at least 23 hr in plastic tubes, but loss of AZM-d_5 signal was observed on glass surface overnight, and much more significant loss of other IS's were observed on glass surface in a few hours. **IS solutions should be prepared and stored in plastic tubes**.

Sample Preparation

<u>Calibration Curve</u>

One set of plasma calibration standards [2/2/1/0.5, 5/5/2.5/1.25, 10/10/5/2.5, 20/20/10/5, 50/50/25/12.5, 100/100/50/25, 200/200/100/50, 500/500/250/125, and 1000/1000/500/250 ng/mL for AZM, HCQ, DHCQ and BDCQ,] are prepared on the day of use according to Table 1.

Condition wells in a HLB micro-elution plate with 200 uL MeOH and drain under vacuum. Add 200 µL water and drain under vacuum again to finish conditioning.. Note: avoid over-drying by using vacuum pressure as low as possible (2-5 mmHg). Empty waste collection plate. Add 40 µL 0.1 M NaOH to each conditioned well, and follow with 20.0 µL of each calibration standard. Aliquot 20.00 µL blank plasma into two wells and labeled them as *blank* and *double blank*. Using a repeat pipettor, add 20 µL internal standard (100/40 /20/20 ng/mL AZM-d_5/HCQ-d_4/DHCQ-d_4/BDCQ-d_4) to all wells except the *double blank*. Mix briefly by shaking the plate and drain slowly under mild vacuum (2-5 mmHg). Once drained, rinse wells twice, once with 200 µL water, and once with 200 µL 10% MeOH; remember to use gentle vacuum (~5 mmHg) each time. Remove HLB plate and wipe the plate tips with Kimwipe. Place a collection plate underneath, elute the HLB SPE plate by adding 25 µL MeOH containing 0.5%FA into each well, drain, and repeat adding and draining one more time. The collected sample volume is ~40 µL. Remove the collection plate and add 150 µL water into each collected sample by 12-channel pipette and mix by pipetting 3 times. Place the collection plate in autosampler. Injection volume is 1.00 µL.

Study and QC samples

Study and QC samples are removed from the freezer and allowed to thaw at room temperature and vortex mixed. Aliquots of 20.0 µL are transferred into the conditioned HLB SPE plate wells and processed as described in the calibration curve section.

Samples above the quantitation limit

If analyzed samples are above the quantitation limit, reprocess them by diluting 20.0 µL of each sample into individual 180 µL blank plasmas. Vortex-mix at 2000 rpm for at least 10 sec. These diluted samples are then processed as described above.

Summary of sample preparation:

1. Wash HLB micro-elution 96-well plate with 200µL MeOH and 200µL water.
2. Add 40 µL 0.1M NaOH to each well.
3. Aliquot 20.0 µL plasma standards or blank plasma, thawed QC controls and unknown samples, into the plate wells.
4. Add 20.0 µL combined internal standard (100 ng/mL AZM-d_5, 40 ng/mL HCQ-d_4, 20 ng/mL DHCQ-d_4, and 20 ng/mL BDCQ-d_4 in 50% MeOH). Mixed briefly, and then drain the wells.
5. Wash with 200 µL water and 200 µL 10% MeOH.
6. Elute the wells with 25 µL MeOH containing 0.5%FA eluent and drain the wells with gradually increased vacuum. Repeat this step once more with another 25 µL eluent.
7. Add 150 µL water into each well with 12-channel pipette and mix by pipetting 3 times.

Chromatography Conditions

LC conditions: Flow rate, 0.5 mL/min; gradient elution: 20% solvent B (0-0.2min), from 20 to 50% B (0.2- 1.5 min), 50-90% B (1.5 -1.6 min), from 90 to 100% B (1.6-2.0 min), 100% B (2.0-2.5 min), from 100 to 20% B (2.5-2.6 min), and 20% B (2.6-3.5 min). injection delay 0.9 min.
Retention times for analytes and their respective I.S. are: 0.92 min (HCQ). 0.79 min (DHCQ), 0.78 min (BDCQ), and 1.84 min (AZM).

Mobile phase: Solvent A is 0.05%TFA in water; solvent B is 0.05% TFA in acetonitrile.

Needle wash solvent: 85% MeCN with 0.1%FA.

Column: PFP column (50 × 2.0 mm, 3 µm, Agilent Tech.) used at room temperature.

Detector: Sciex API5000 tandem mass spectrometer

Volume of injection: 1 μL into a 50 μL loop.

MS-MS Parameters

Ionization: ElectroSpray ionization in positive mode (ESI^+)
Ion source temperature: 500 °C
Curtain gas (CUR): 25 psi (Nitrogen)
Nebulizer Gas (Gas 1): 50 psi (zero air)
Gas 2: 40 psi
Ion spray voltage: 1250 v
Collision-Activated Dissociation (CAD) Gas: 12

MRM ion pair	DP	EP	CE	CXP
AZM 749.6/591.6	50	10	40	39
AZM-d5 754.6/596.6	50	10	40	39
HCQ 336.1/247.1	50	10	29	16
HCQ-d_4 342.1/253.1	50	10	29	16
DHCQ 308.1/179.1	50	10	31	16
DHCQ-d4 314.1/181.1	50	10	31	16
BDCQ 264.1/179.1	50	10	30	16
BDCQ-d_4 270.1/181.1	50	10	30	16

Note: DP is declustering potential, applied to the orifice plate. EP is entrance potential. CE is collision energy. CXP is collision cell exit potential.
Resolution is set at unit at Q1 and unit at Q3. Scan dwell time is 50 ms for each transition, pause between mass ranges is 5 ms. The MS scan window is 0.5-2.5 min (the LC eluent was directed to MS source at 0.5 min and to the waste line at 2.5 min).

Calibration determination and calculation for quality controls and unknowns
All samples are stored at -70°C until prepared for analysis.
For calibration curves, nominal spiked concentrations and peak area ratios of analyte/IS are fitted by linear regression weighted by 1/x, except for AZM using quadratic regression with $1/x^2$ weighting factor (Analyst® 1.6.3). Concentrations are calculated from the regression parameters using peak area ratios.

$$\text{Accuracy} = \frac{\text{Calculated concentration} \times 100}{\text{Theoretical concentration}}$$

Note: One set of calibrators was processed. The calibrators were injected at the beginning of the batch run.

System Suitability per batch

At the beginning of each run day a M- or H-QC sample is injected onto the column repeatedly (3-6 injections) to determine detector response, chromatography quality and retention time. The coefficient of variation (CV%) of peak area ratio for 3 continuous injections should be <10% before starting the batch run. The QC used for system suitability is separate from the QC used in the analytical run. The retention time can be varied from day to day depending on the column condition, LC pump efficiency and system pressure. Within a batch of run, retention times for analytes and IS should be consistent (± 0.05 min).

Acceptance Criteria

1) To verify stock and working solutions, the %difference of two solutions from separately weighed standard drugs must be <10% (preferably <5.0%) to be acceptable. As the limited source of the metabolites DHCQ and BDCQ, the stock solutions are prepared from the purchased compounds with exact weights. QC stock solutions may be the same as calibrator standard stocks if the accuracy of stocks is verified, either from a certified stock solution or by a separately weighed stock. Newly prepared plasma calibrators should be verified with Qc samples that are either prepared freshly from separate stock/working solution or verified previously.

2) Calibrators were injected in the beginning of each run, the peak response in blank plasma sample should be <20% of LLOQ peak for analytes and <5.0% for the IS. For all calibrators, except LLOQ, accuracy (% of theoretical value) must have values within 85.0-115% (including 85.0% and 115%) when compared to the theoretical concentration. The LLOQ % deviation must be within 80.0-120% when compared to the theoretical value. Calibrators that do not meet the criteria will be omitted. If any point is excluded the calibration curve should be recalibrated. A minimum of 7 calibrators must be included to accept the run in this assay. Correlation coefficient (r) should be ≥ 0.995 (0.990 for r^2).

3) A minimum of 4 out of 6 quality controls (low, medium, high) must have their accuracy value within 85.0-115% of the theoretical. A minimum of 50% QCs at each level must be within 85.0-115% of the theoretical.

4) For runs to be acceptable, a maximum of two calibrators below ULOQ are allowed to be masked/dropped but they cannot be consecutive. The run should be rejected if the calibration curve is truncated because ULOQ fails.

5) If a specimen is rejected from the run due to above ULOQ for one or more analytes, re-analysis is performed with proper dilution when applicable. The assay is validated for up to 10-fold dilution. Results from re-analysis are only reported for the analytes above ULOQ.

6) IS signal should be consistent throughout the run with all samples within ±25% of mean IS signal. Samples falling out of this range should be investigated and re-analyzed if necessary.

7) If the run does not meet acceptance criteria for one or more analytes, re-analysis of the whole run is performed and results from re-analysis are only reported for those analytes failed in the initial analysis.

8) Integration of peak should be checked for each injection. Reintegration by adjusting bunching factor or smoothness for the whole run is acceptable. Reintegration for individual peaks is only allowed in the cases of missed or partial peak integration due to retention time, peak shape, or baseline variation, and chromatograms before and after reintegration should be documented if manual integration were used.

9) If multiple batches of samples are processed and analyzed in a whole run, one or more sets of QCs should be processed in each batch of samples. The total number of QCs in a batch must be at least 5% of the number of unknown samples.

10) Data is reported in an excel spreadsheet following review by a QC person based on the established "Run Acceptance Criteria" and the laboratory post-run checklist for LC-MS/MS analysis. The excel spreadsheet contains sample information sent during sample shipment.

Limits

(1). SPE plate already used for extraction of AZM/HCQ/DHCQ/BDCQ cannot be reused.

(2), PFP column performance is varied if the PFP column is from different vendors, or even from the same vendor but different lots. It may be necessary to condition the PFP column using mobile phases for an extended time. The retention time of analytes may be changed when new column is used. This is considered acceptable as long as retention time is consistent within the run. In addition, column should be washed with 80% isopropanol overnight when splitted or tailing peaks are observed, typically after 5-10 days of use.

(3).There is carryover for HCQ and DHCQ in this assay that is typically less than 20% of LLOQ peak. To monitor carryover, a double blank injection should be inserted after ULOQ (Cal 9) and QC-H. In the case of more than 20% carryover (never happen during validation), reinjection of samples near LLOQ level is required if the sample is analyzed following an H-QC or a sample near H-QC level.

References

1 Parnham, M. J. *et al.* Azithromycin: mechanisms of action and their relevance for clinical applications. *Pharmacol Ther* **143**, 225-245, doi:10.1016/j.pharmthera.2014.03.003 (2014).

2 Chico, R. M. & Chandramohan, D. Azithromycin plus chloroquine: combination therapy for protection against malaria and sexually transmitted infections in pregnancy. *Expert Opin Drug Metab Toxicol* **7**, 1153-1167, doi:10.1517/17425255.2011.598506 (2011).

3 Supattanapong, S. & Konsil, J. Solid phase extraction and high performance liquid chromatography for the determination of azithromycin in human plasma. *Southeast Asian J Trop Med Public Health* **39**, 978-987 (2008).
4 Gautret, P. *et al.* Hydroxychloroquine and azithromycin as a treatment of COVID-19: results of an open-label non-randomized clinical trial. *Int J Antimicrob Agents*, 105949, doi:10.1016/j.ijantimicag.2020.105949 (2020).
5 Sperber, K., Hom, C., Chao, C. P., Shapiro, D. & Ash, J. Systematic review of hydroxychloroquine use in pregnant patients with autoimmune diseases. *Pediatr Rheumatol Online J* **7**, 9, doi:10.1186/1546-0096-7-9 (2009).
6 Rempenault, C. *et al.* Clinical and Structural Efficacy of Hydroxychloroquine in Rheumatoid Arthritis: A Systematic Review. *Arthritis Care Res (Hoboken)* **72**, 36-40, doi:10.1002/acr.23826 (2020).
7 Liu, J. *et al.* Hydroxychloroquine, a less toxic derivative of chloroquine, is effective in inhibiting SARS-CoV-2 infection in vitro. *Cell Discov* **6**, 16, doi:10.1038/s41421-020-0156-0 (2020).
8 Fan, H. W. *et al.* Pharmacokinetics and Bioequivalence Study of Hydroxychloroquine Sulfate Tablets in Chinese Healthy Volunteers by LC-MS/MS. *Rheumatol Ther* **2**, 183-195, doi:10.1007/s40744-015-0012-0 (2015).
9 Colson, P., Rolain, J. M., Lagier, J. C., Brouqui, P. & Raoult, D. Chloroquine and hydroxychloroquine as available weapons to fight COVID-19. *Int J Antimicrob Agents*, 105932, doi:10.1016/j.ijantimicag.2020.105932 (2020).
10 Mehnert, J. M. *et al.* A phase I trial of MK-2206 and hydroxychloroquine in patients with advanced solid tumors. *Cancer Chemother Pharmacol* **84**, 899-907, doi:10.1007/s00280-019-03919-x (2019).
11 Soichot, M. *et al.* Development, validation and clinical application of a LC-MS/MS method for the simultaneous quantification of hydroxychloroquine and its active metabolites in human whole blood. *J Pharm Biomed Anal* **100**, 131-137, doi:10.1016/j.jpba.2014.07.009 (2014).
12 Fan, J. *et al.* Connecting hydroxychloroquine in vitro antiviral activity to in vivo concentration for prediction of antiviral effect: a critical step in treating COVID-19 patients. *Clinical Infectious Diseases*, doi:10.1093/cid/ciaa623 (2020).
