# Supplementary material for: Development and validation of an LC-MS/MS method for determination of hydroxychloroquine, its two metabolites, and azithromycin in EDTA-treated human plasma
Source: PLoS One. 2021 Mar 5;16(3):e0247356. doi: 10.1371/journal.pone.0247356 (PMC7935301; doi:10.1371/journal.pone.0247356)
Supplement: Attachment — Submitted filename: ResponseToReviewers.docx [file pone.0247356.s006.docx]

Response to reviewers’ comments on manuscript entitled “ A validated LC-MS/MS method for determination of azithromycin, hydroxychloroquine, and its metabolites desethylhydroxychloroquine and bisdesethylchoroquine in human plasma” (PONE-D-20-26989).

Comments from the Editor’s office.

*1. Please ensure that your manuscript meets PLOS ONE's style requirements, including those for file naming*.

Response: The manuscript was revised with updated format and style.

*2. For reproducibility purposes please clearly specify in your methods section the source of the blood and plasma used in your study (eg. brand, product number)*

 Response: blank blood and plasma sources are now added.

*3. Thank you for stating in your Funding Statement:*

*"This work was partially supported by the National Institutes of Health (NIH) through AIDS clinical Trials Group (ACTG), grant number 1UM1 AI068636 (F.A.). URL:* [*www.NIH.gov*](https://urldefense.proofpoint.com/v2/url?u=http-3A__www.NIH.gov&d=DwQGaQ&c=iORugZls2LlYyCAZRB3XLg&r=IkxXbyXaHsrnvExdn1fxsAErVwUCspeoHLMb3_z93fo&m=AeaYtdpeJhAIDUw_jJyIMA4WE2ZXr40yo99ppVq3t1o&s=qniSD56CYAmRxFtMkwndi4TisHRpEGVAOes2nVjZHmA&e=)*. The funder plays no role in the study or prepataion of the manuscript."*

*Please provide an amended statement that declares *all* the funding or sources of support (whether external or internal to your organization) received during this study, as detailed online in our guide for authors at* [*http://journals.plos.org/plosone/s/submit-now*](about:blank)*.  Please also include the statement “**There was no additional external funding received for this study.” in your updated Funding Statement.*

*Please include your amended Funding Statement within your cover letter. We will change the online submission form on your behalf.*

 Response: amended funding statement was included in this letter.

*4. We note that you have included the phrase “data not shown” in your manuscript. Unfortunately, this does not meet our data sharing requirements. PLOS does not permit references to inaccessible data. We require that authors provide all relevant data within the paper, Supporting Information files, or in an acceptable, public repository. Please add a citation to support this phrase or upload the data that corresponds with these findings to a stable repository (such as Figshare or Dryad) and provide and URLs, DOIs, or accession numbers that may be used to access these data. Or, if the data are not a core part of the research being presented in your study, we ask that you remove the phrase that refers to these data.*

 Response: We addressed the “data not shown” issues by providing the data in Supporting S2 Figure and S2 Tables.

__________________
Reviewers' comments:

Reviewer #1: Comments to Author:

*1. Abstract should be limited to 200 words*

Response: The abstract was truncated but still over 300 words limit. We deem exception should be granted in this case as this is a comprehensive assay with 4 analytes.

*2. Keywords should not be the repetitions of the title words, please find such words which are not in the title, this way search engines of the web will find your manuscript with higher probability.*

Response: additional key words are included: malaria; solid-phase extraction;

*3. Suggestions for improvements in the Title*

Response: The title is updated as follows: Development and validation of an LC-MS/MS method for determination of hydroxychloroquine, its two metabolites, and azithromycin in EDTA-treated human plasma.

*4. The structure of scientific publication should include the general chapters (Introduction, Materials and Methods, Results, Discussion, Conclusion). Please follow instructions on journal webpage. Conclusion chapter is missing.*

Response: we have revised the conclusion section (4), and discussion was incorporated in the results section.

*5. Recommendations for future studies are needed in the conclusion section. Kindly provide strong recommendations for future researches.*

Response: In Conclusion section, we discussed the potential use of the method for studies other than Covid-19. We also added the following sentences:

“For it requires only a small sample volume, our method can be used for pediatric studies where sample volume is limited, and it can be coupled with capillary tube sampling by a finger prick or more advanced microsampling techniques such as Seventh Sense Tap™ to facilitate clinical studies. With the highly sensitive LC-MS/MS system, our method may be modified for dried blood spot samples.”

*6. Some citations are missing from the References section.*

Response: The citations in text can all be found in references section

*7. Some references are not cited in the text.*

Response: All 37 references are now cited in the text using ENDNote program.

*8. Formatting does not match journal criteria. (e.g. References section; section title spacing; paragraph indents)
The paper generates the following kinds of data.
1. Only 20 µL plasma sample by volume is needed for simultaneous quantitation of AZM, HCQ, DHCQ, and BDCQ.
2. The run time is 3.5 min which is fast turnaround time.
3. Statistical measurements are missing in whole of the data which should be added in the manuscript.
However, before I can recommend its publication, the authors should address the following questions
Some questions to author
1. The %differences from controls are 3.8% and -2.9% for AZM, 1.3% and 2.7% for HCQ, -1.9% and -0.9% for DHCQ, and -2.0% and -5.7% for BDCQ. Can author explain what would be the structural changes?
2. Why the study is important, what benefits readers can get from this study?*

Response: Formatting was updated. The % differences were calculated from the measured control QC-L and QC-H concentrations. The data to calculate the % differences are now provided in Supporting S2 tables.

The method was developed to support potential clinical studies. Readers in the field of COVID-19, malaria, and inflammatory diseases using these drugs may be interested in the method and drug stability data. The small sample volume used in our method will benefit pediatric studies as stated in the conclusion section now. Furthermore, the approaches for method development are exemplary for readers in bioanalysis field,.

Reviewer #2: 
*1. Page 5, line 3: There are two periods after [21].*

Response: The extra period is removed now.

*2. Page 6, Figure 1: I recommend having the same relative geometry of the rings in the structure for the last 6 compounds to aid in visualizing the differences between structures*.

Response: The Figure 1 is updated.

*3. Page 6, Section 2.2: PFP should be defined for the more general reader.*

Response: It is now defined as follows: pentafluorophenyl (PFP)

*4. Page 7, section 2.4: HLB should be defined for the more general reader.*

Response: It is now defined as follows: Hydrophilic lipophilic balance (HLB) solid phase extraction.

*5. Page 5 and 22-23: These equations should be reformatted and numbered using the recommended standards of the journal.*

Response: The equations are reformatted and numbered now.

*6. Page 19, end of Room Temperature Stability in Plasma section: (Table] should be replaced with (Table 5).*

Response: It is now corrected.
